# Scientific data from precipitation driver response model intercomparison project

**DOI:** 10.1038/s41597-022-01194-9

**Published:** 2022-03-30

**Authors:** Gunnar Myhre, Bjørn Samset, Piers M. Forster, Øivind Hodnebrog, Marit Sandstad, Christian W. Mohr, Jana Sillmann, Camilla W. Stjern, Timothy Andrews, Olivier Boucher, Gregory Faluvegi, Trond Iversen, Jean-Francois Lamarque, Matthew Kasoar, Alf Kirkevåg, Ryan Kramer, Longbo Liu, Johannes Mülmenstädt, Dirk Olivié, Johannes Quaas, Thomas B. Richardson, Dilshad Shawki, Drew Shindell, Chris Smith, Philip Stier, Tao Tang, Toshihiko Takemura, Apostolos Voulgarakis, Duncan Watson-Parris

**Affiliations:** 1 CICERO - Center for International Climate Research, Oslo, Norway; 2 Faculty of Environment, University of Leeds, Leeds, UK; 3 Met Office Hadley Centre, Exeter, UK; 4 Institute Pierre‐Simon Laplace, Sorbonne Université/CNRS, Paris, France; 5 Center for Climate System Research, Columbia University, New York, NY USA; 6 NASA Goddard Institute for Space Studies, New York, USA; 7 Norwegian Meteorological Institute, Oslo, Norway; 8 National Center for Atmospheric Research, Boulder, CO USA; 9 Leverhulme Centre for Wildfires, Environment and Society, Department of Physics, Imperial College London, London, UK; 10 Climate and Radiation Laboratory, NASA Goddard Space Flight Center, Greenbelt, MD 20771 USA; 11 Universities Space Research Association, 7178 Columbia Gateway Drive, Columbia, MD 21046 USA; 12 Northwest Institute of Nuclear Technology, Xi’an, China; 13 Institute of Meteorology, Universität Leipzig, Leipzig, Germany; 14 now at Pacific Northwest National Laboratory, Richland, WA USA; 15 Nicholas School of the Environment, Duke University, Durham, NC USA; 16 Department of Physics, University of Oxford, Oxford, UK; 17 School of Environment, Yale University, New Haven, CT USA; 18 Research Institute for Applied Mechanics, Kyushu University, Fukuoka, Japan; 19 School of Environmental Engineering, Technical University of Crete, Chania, Greece

**Keywords:** Climate change, Atmospheric science

## Abstract

This data descriptor reports the main scientific values from General Circulation Models (GCMs) in the Precipitation Driver and Response Model Intercomparison Project (PDRMIP). The purpose of the GCM simulations has been to enhance the scientific understanding of how changes in greenhouse gases, aerosols, and incoming solar radiation perturb the Earth’s radiation balance and its climate response in terms of changes in temperature and precipitation. Here we provide global and annual mean results for a large set of coupled atmospheric-ocean GCM simulations and a description of how to easily extract files from the dataset. The simulations consist of single idealized perturbations to the climate system and have been shown to achieve important insight in complex climate simulations. We therefore expect this data set to be valuable and highly used to understand simulations from complex GCMs and Earth System Models for various phases of the Coupled Model Intercomparison Project.

## Abstract (summary)

**Table:** 

Measurement(s)	Climate response
Technology Type(s)	Climate models
Sample Characteristic - Environment	The climate system

## Background & Summary

The Precipitation Driver Response Model Intercomparison Project (PDRMIP)^1^ was launched in November 2013 in an effort to improve our insight on how various mechanisms that perturb the planetary radiation balance induce precipitation changes. This includes the most important anthropogenic climate drivers such as greenhouse gases and aerosols as well as natural changes in the solar incoming radiation.

The motivation for PDRMIP arose from climate model simulations showing that the global and annual mean precipitation changes occurring on a fast time scale, of the order of months to a few years, depend on the atmospheric absorption and are strongly dependent on climate drivers, whereas the slow response, on order of decades to centuries, is dependent on the temperature change and independent of climate drivers^2,3^. The physical reason for these relationships is energy constraints^4–6^. The link between precipitation changes and climate sensitivity due to perturbation of the radiation balance by a climate driver is strong, but the diversity between climate models is not fully settled^7^.

PDRMIP includes idealised experiments of large and abrupt changes in all major greenhouse gases and aerosols. The experiment setup allows for diagnosing perturbations to the radiation balance and the precipitation changes both on a fast and a slow timescale. Twelve modelling groups have submitted PDRMIP results. Analyses of the PDRMIP results are being conducted by groups contributing to PDRMIP as well as groups outside the core PDRMIP modelling groups.

The PDRMIP data are valuable in several aspects, their main purpose was to contribute to the understanding of precipitation changes, but the data also yield insights into radiative forcing, climate feedbacks and surface temperature changes. The results from PDRMIP have so far given improved knowledge of how the climate drivers influence the hydrological sensitivity and extreme precipitation on a regional and global scale^8–16^. One main result from the PDRMIP analysis is that black carbon in the atmosphere has a weak surface temperature response caused by a strong negative rapid adjustment^17,18^. Two studies have provided important knowledge on the effective radiative forcing (ERF) concept^19,20^. The PDRMIP data have in several studies been used to understand the importance of different climate drivers in complex Coupled Model Intercomparison Project Phase 5 (CMIP5)^21^ simulations^12,13,22,23^. An aim of this study is that PDRMIP data will be used to understand complex general circulation model simulations of the climate system by analysing the data in new ways or in context of CMIP6^24^ simulations.

## Methods

Table S1 shows an overview of the models contributing to PDRMIP and the experiments. PDRMIP has three sets of simulations: six core experiments, five regional experiments and six Phase 2 experiments. The core experiments cover the two main greenhouses gases in terms of anthropogenic climate change, two of the major atmospheric aerosol components and natural changes of solar irradiance. To achieve a substantial signal to the climate system, large perturbations have been performed, by doubling the CO_2_ concentration (CO2x2), tripling the CH_4_ concentration (CH4x3), multiplying the anthropogenic sulphate abundance by five (Sulx5) and the black carbon (BC) by ten (BCx10), and increasing the solar irradiance at the top of the atmosphere by 2% (solar). The regional experiments include perturbations to BC and sulphate in Europe and Asia. The PDRMIP Phase 2 experiments have been performed so that all the main anthropogenic climate forcing mechanisms during the industrial era are covered. The changes to halocarbons have been from present abundances to 5 ppb given experiment names CFC11 and CFC12, N_2_O has been changed from 316 ppb to 1 ppm (experiment name N2O1p) and the ozone abundance in the troposphere has been multiplied by five (ozone). The land use changes caused by agriculture have been impossible to scale and therefore the change over the industrial era has been considered (lndus). Observations have shown that most global aerosol models have too high abundance in the middle and upper troposphere and therefore a too long lifetime for BC^25,26^. An experiment with short BC lifetime^27^ and BC concentrations multiplied by a factor of ten has thus been performed (bcslt). The anthropogenic aerosol distribution in BCx10 and Sulx5 is either from fixed concentrations from the multi-model mean of AeroCom Phase II models^28^ or based on emissions. Fixed concentrations from a global aerosol model^27^ are used in bcslt. All the PDRMIP experiments are abrupt perturbations to the Base experiment, which is part of the Core simulations. Table S1 shows that a total of 114 simulations have been performed with the 11 global climate models.

For each of the experiments a fixed sea-surface temperature (fsst) of a minimum of 15 years and a coupled atmosphere-ocean simulation of a minimum of 100 years have been performed. The standard approach in PDRMIP has been to analyse the fsst simulations for years 6–15 and the coupled simulations for years 51–100^15^. A description of the PDRMIP models is given in Table 3 in the PDRMIP overview paper^1^.

The output follows to a large extent the protocol for a subset of the atmospheric variables in CMIP5 for monthly and daily data. The output protocol for the PDRMIP variables is available at the PDRMIP website (https://www.cicero.oslo.no/en/PDRMIP). A list of available monthly 2-dimensional fields at the surface or top-of-atmosphere (TOA), and 3-dimensional fields for the whole atmosphere, is given in Table S2. Daily fields are available for temperature (mean, maximum and minimum) and precipitation total and convective).

## Data Records

**Fig. 1:**
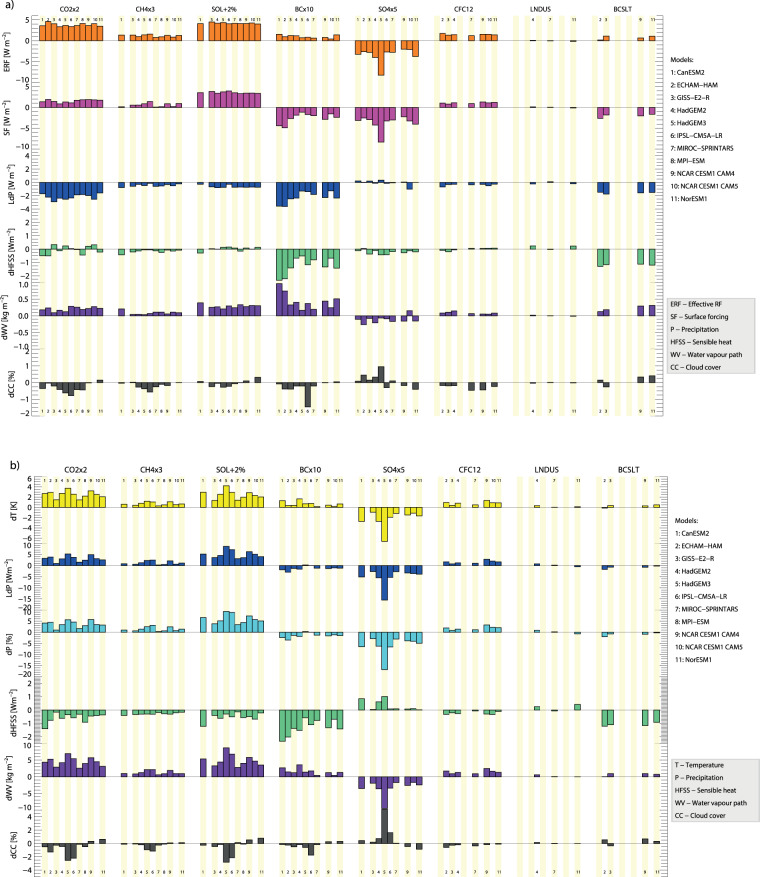
Global and annual mean numbers from eight PDRMIP experiments and eleven models, results from fsst simulations (**a**) and from coupled simulations (**b**).

To illustrate the main PDRMIP results^29^, which are listed in Tables S3–11, this section also includes a figure to describe essential aspects of the PDRMIP simulations with references to detailed PDRMIP studies. Figure 1 shows global and annual mean numbers for core PDRMIP variables for the individual models and eight experiments for fsst and coupled simulations. The figure illustrates the model spread described in earlier PDRMIP publications on differences in temperature changes, such as for a doubling of CO_2_ and in the BC experiment^15,17^. The best model agreement is found for the solar and CFC12 experiments in the fsst simulations. Even though substantial differences often are found in the solar radiative effects in climate models^30^, the ERF and additional main PDRMIP results from the fsst simulations show smaller diversity in the solar experiment than the other core PDRMIP experiments^11,18^. CFC-12 absorbs in a spectral region with small absorption by water vapour in the ‘atmospheric window’, which may contribute to relatively small model diversity for the CFC12 experiment. In the fully coupled simulations (Fig. 1b) the climate sensitivity^31,32^ contributes to the much larger spread in the solar results than in fsst simulations (Fig. 1a).

The differences between results shown in Fig. 1a,b is purely driven by changes in the surface temperature change (climate feedback process) of changes to water vapour and cloud cover, where PDRMIP results show large similarities among the climate drivers per degree global surface warming^13^. Changes in clouds in Fig. 1a are caused by different processes for the climate drivers. For SO_4_ some of the models include aerosol-cloud interactions (see the PDRMIP website with detailed description of the models and the PDRMIP overview publication^1^). For BC and CO_2,_ the cloud changes arise from modifications in the atmospheric vertical profile of temperature (lapse rate) caused by atmospheric heating (difference between the two first rows in Fig. 1a) and may vary by altitude^17^.

Supplementary Fig. 1 shows similar numbers to Fig. 1 for three regional and three Phase 2 experiments. Fewer models have taken part in the Phase 2 experiments than the core experiments, but results are available for as much as eight models in the CFC12 experiment. For the two CFC experiments, CH_4_ and N_2_O no indirect chemical effects are included in the model simulations (see Supplementary Table S12). The CFC11 experiment (shown in Supplementary Fig. 1) has results similar to the CFC12 experiment. Since N_2_O has an atmospheric abundance and absorption in the longwave spectrum more similar to CH4, the changes in precipitation, water vapour, sensible heat in the fsst and coupled simulations between these two greenhouse gases agree better than other greenhouse gases. The Lndus experiment was impossible to scale to achieve a large ERF or temperature response. Only the sensible heat (hfss) from the Lndus experiment has a magnitude comparable to some of the other PDRMIP experiments. All the regional experiments show weak responses compared to BC and sulphate in the core PDRMIP simulations due to much smaller perturbations.

Table S3 and S4 show global and annual multi-model mean results for PDRMIP variables for the base experiment and the difference between the PDRMIP drivers experiments and the base case. The number of models that have performed the experiments are given in parenthesis. Values are given for the fsst and the coupled simulations. Table S3 provides the TOA (t) and surface (s), upward (u) and downward (d), shortwave (s) and longwave (l) radiative fluxes (r) as well as the surface fluxes of sensible (hfss) and latent heat (hfls). The rsut variable is thus the upward shortwave radiative flux at TOA. The ERF shown in Fig. 1a is the net downward radiative flux at TOA and is rsdt – rsut – rlut. The change in the atmospheric radiative cooling is fundamental for global changes in the precipitation^1,2,4,15^. The fast change in atmospheric radiative cooling is derived as the difference between the surface forcing and ERF, where the former is net change in downward longwave and shortwave radiative fluxes (rlds – rlus + rsds – rsus). For BCx10 the change in the fsst atmospheric radiative cooling is particularly large^15^ and with a negative sign it gives an atmospheric radiative heating and thus a reduction in the precipitation in the fsst simulations. Similarly, the radiative cooling in the coupled simulations can be derived from the difference between the net downward surface and TOA radiative fluxes, where an increase in surface temperature will lead to a radiative cooling.

In Table S4 the results for the most important meteorological variables are provided. The variables surface air temperature (tas), precipitation (pr), column water vapour (prw), and cloud cover (clt) are also shown in Fig. 1. The global mean precipitation equals the global mean evaporation (evspsbl). The latent heat (hfls) is the energy flux of the evaporation. The release of latent heat in the atmosphere through condensation must globally balance the radiative cooling subtracted the sensible heat (hfss).

Tables S5–11 show global and annual results for individual PDRMIP models for the same core variables as given as multi-model mean values in Table S3 and S4. Additional variables and results from Phase 2 experiments are shown in Supplementary Tables. Whereas Tables S3 and S4 provide differences between the base and the PDRMIP driver experiments, Tables S5–11 show the actual global and annual mean numbers for all PDRMIP simulations. The PDRMIP website (see Usage section) provides data with more digits relative to Tables S3–11 and standard deviations among the PDRMIP models for Tables S3 and S4.

## Technical Validation

The energy budget with surface and TOA fluxes given in the base experiments in Table S3, S5, S6, and S7 is well within the uncertainties for the multi-model mean in the current knowledge and quantification of these numbers^33,34^, and only with some very few exceptions for the individual models. The differences between the estimates of the Earth energy budget are largest for surface fluxes^33,34^, and the only case with more than one model having a radiative flux outside the range of the two most reliable estimates is for the longwave upwelling at the surface (rlus).

**Fig. 2:**
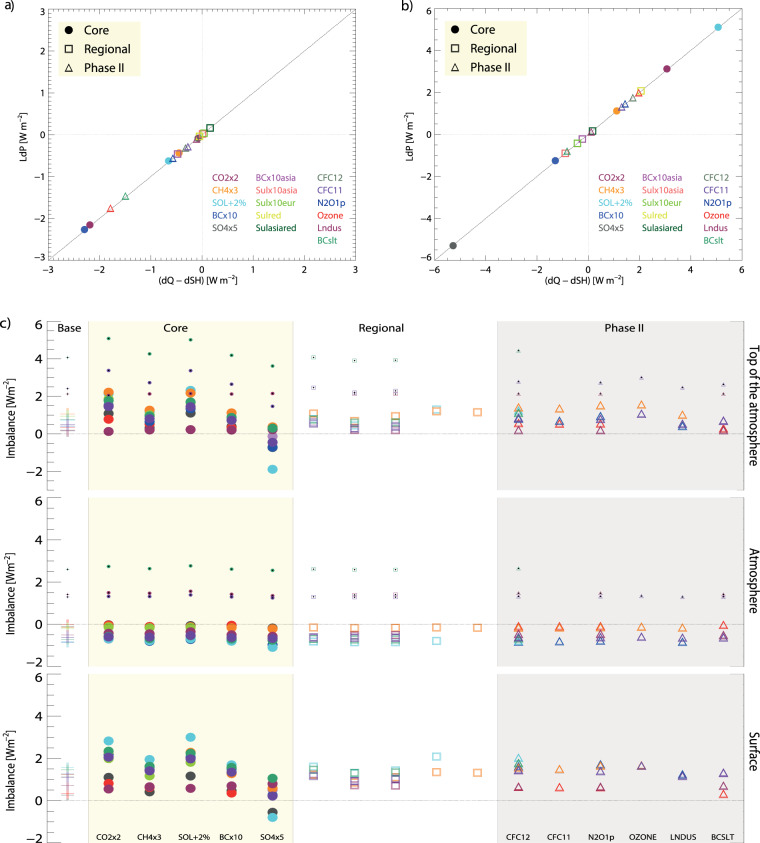
The PDRMIP multi-model mean of precipitation in energy fluxes (W m^−2^) is shown as a function of change in the atmospheric radiative cooling and the reduction of surface sensible heat for all PDRMIP experiments in the fsst simulations (**a**) and coupled simulations (**b**). The TOA, atmospheric and surface imbalance in the Base and PDRMIP perturbation experiments (**c**). Radiative imbalance at top of the atmosphere, in the atmosphere and at the surface for all PDRMIP experiments and models in the coupled atmosphere and ocean simulations. Small symbols are included for reported values for three of the models whereas regular symbols take into account difference between the top model layer and TOA.

The individual PDRMIP models in the five core PDRMIP perturbation simulations are shown to conserve energy by precipitation changes in energy fluxes being equal to the sum of the changes in the radiative cooling and reduction in sensible heat from the surface to the atmosphere in the fsst and coupled simulations^12^. Fig. 2a,b show that the energy budget is closed for the model mean in all the PDRMIP experiments. The change in precipitation for BC and sulphate is different relative to other drivers with strong reduction for BC in Fig. 2a and more modest changes in the coupled climate simulations (Fig. 2b). For sulphate this is the opposite with strong change in the coupled climate simulation. This is an important result from the PDRMIP dataset and is also illustrated in Fig. 1 where BC has a strong difference between TOA and surface fluxes in Fig. 1a and a relatively small temperature change in Fig. 1b. Again, this is the opposite for sulphate compared to BC and several PDRMIP studies have investigated this in more detail^11,13–15^.

Figure 2c shows that the PDRMIP models for the Base and perturbation simulations mostly have an imbalance at the top of the atmosphere (TOA), atmospheric and surface within 2 Wm^−2^, but 2-3 models have imbalances of the order of 4 Wm^–2^. This larger model imbalance for some models is due to account of changes between the highest model layer and the TOA. The correction of this effect is shown with standard symbols and the reported fluxes in small symbols.

## Usage Notes

**Fig. 3:**
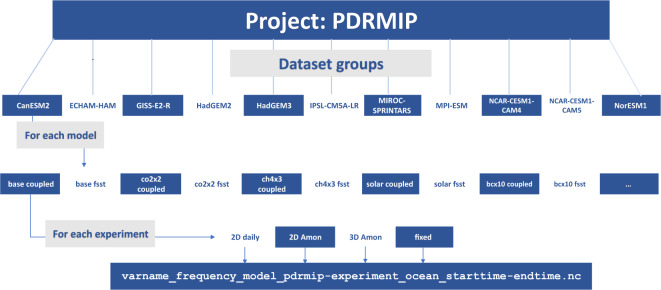
A schematic representation of the PDRMIP data. The data are available through the data storage World Data Center for Climate (WDCC) https://www.dkrz.de/up/systems/wdcc. The folder with the address given on top of the schematic is the main folder for the data. It contains separate subfolders for each model. In each of the model subfolders, there are separate folders for the fsst and coupled ocean conditions for each experiment. For each experiment there are subfolders for 2-dimensional daily data, 2-dimensional monthly data, 3-dimensional monthly data, and model fixed variables. In these folders data for each separate variable can be found, with names according to the formatting detailed in the lowermost box in the figure.

The PDRMIP data are available through the World Data Center for Climate (WDCC) https://www.dkrz.de/up/systems/wdcc with 10.26050/WDCC/PDRMIP_2012-2021^29^. The data are stored and organised according to the schematic in Fig. 3, with data for each variable, time frequency (day or Amon), model, experiment and ocean configuration in a separate file. The file name also indicates what time period the data span. The PDRMIP website (https://www.cicero.oslo.no/en/PDRMIP) has a python script for reading the PDRMIP data.

## Supplementary information

Supplementary Material

## Data Availability

A code to extract the PDRMIP data is available at the storage of the data (see usage section) where all the PDRMIP data are freely available. The PDRMIP data are available through the World Data Center for Climate (WDCC) https://www.dkrz.de/up/systems/wdcc with 10.26050/WDCC/PDRMIP_2012-2021^29^.
