# Supplementary material for: Scientific data from precipitation driver response model intercomparison project
Source: Sci Data. 2022 Mar 30;9:123. doi: 10.1038/s41597-022-01194-9 (PMC8967886; doi:10.1038/s41597-022-01194-9)
Supplement: Supplementary file 1 — Supplementary Material [file 41597_2022_1194_MOESM1_ESM.pdf]

Supplementary for paper *Scientific data from Precipitation Driver Response Model Intercomparison Project* by G. Myhre et al.

a)

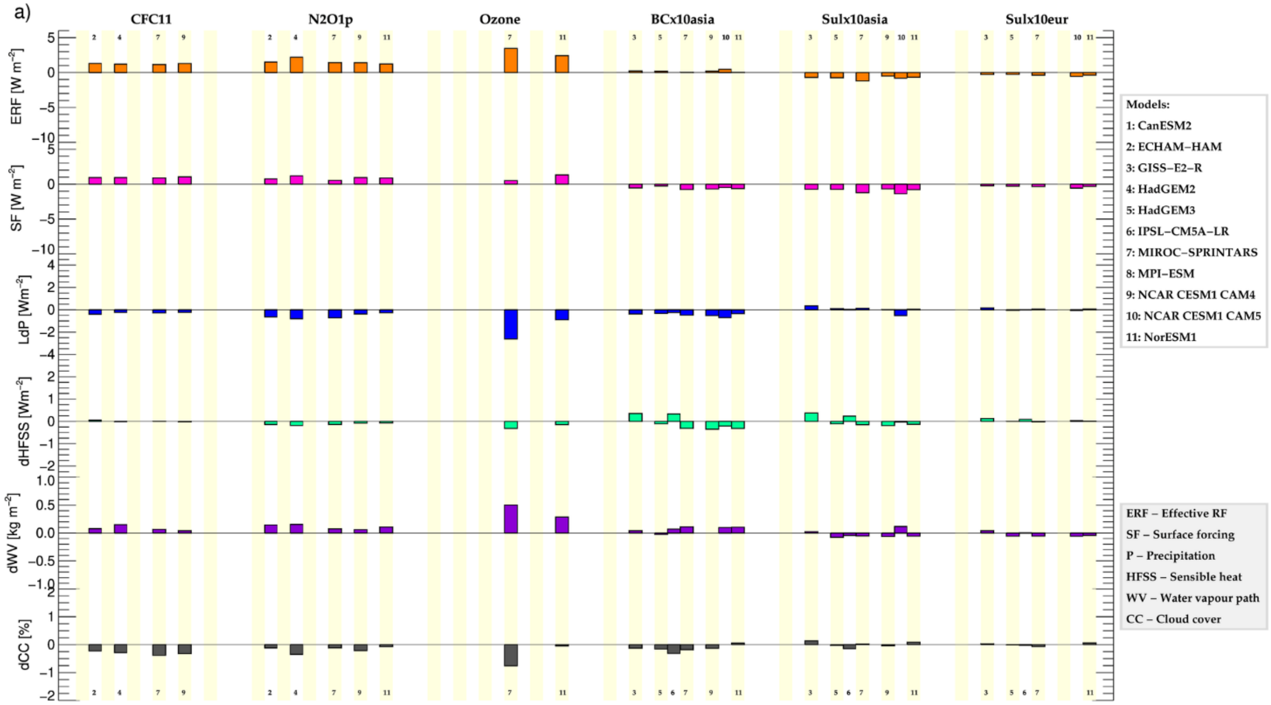

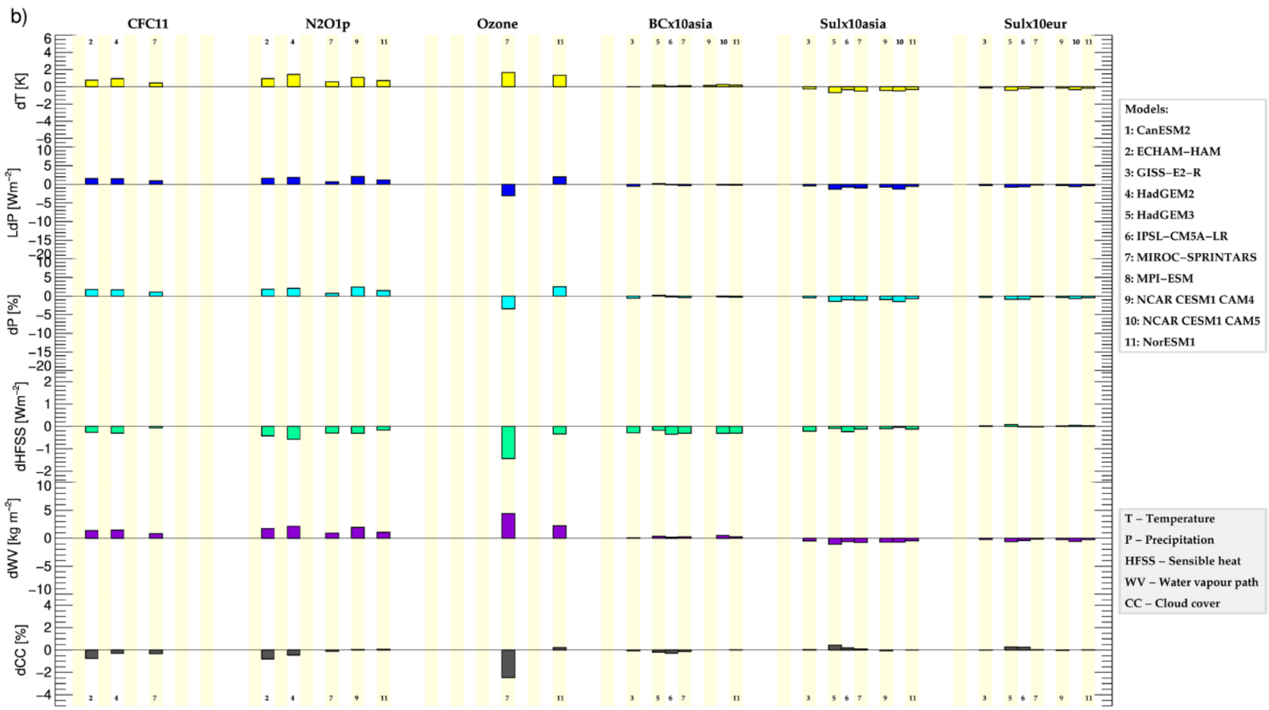

Figure S1: Global and annual mean numbers from six PDRMIP experiments and eleven models, results from fsst simulations a) and from coupled simulations b).

Table S1: Overview of available PDRMIP data (experiments conducted per model). Two models required a new Base simulation due to slightly different model versions have been used, denoted here as Base2. In ECHAM-HAM a base4 was required in the bcslt experiment because the abundance of BC will be different from base when deposition processes are changed.

Experiments	CanESM2	MPI-ESM	NorESM1	CESM1-CAM4	CESM1-CAM5	MIROC-SPRINTARS	HadGEM2	HadGEM3	GISS-E2-R	IPSL-CM5A-LR	ECHAM-HAM
**Core**											
Base	✓	✓	✓	✓	✓	✓	✓	✓	✓	✓	✓
CO2x2	✓	✓	✓	✓	✓	✓	✓	✓	✓	✓	✓
CH4x3	✓	✓	✓	✓	✓	✓	✓	✓	✓	✓	X
Solar	✓	✓	✓	✓	✓	✓	✓	✓	✓	✓	X
BCx10	✓	X	✓	✓	✓	✓	✓	✓	✓	✓	✓
Sulx5	✓	X	✓	✓	✓	✓	✓	✓	✓	✓	X
**Regional**											

BCx10asia	X	X	✓	✓	✓	✓	X	✓	✓	✓	X
Sulx10asia	X	X	✓	✓	✓	✓	X	✓	✓	✓	X
Sulx10eur	X	X	✓	✓	✓	✓	X	✓	✓	✓	X
Sulred	X	X	X	X	X	✓	X	✓	X	X	X
Sulasiared	X	X	X	X	X	✓	X	X	X	X	X
**Phase 2**											
Base2	X	X	X	X	X	X	✓	X	X	X	✓
cfc12	X	X	✓	✓	✓	✓	✓	✓	✓	X	✓
cfc11	X	X	X	✓	X	✓	✓	X	X	X	✓

n2o1p	✗	✗	✓	✓	✗	✓	✓	✗	✗	✗	✓
Ozone	✗	✗	✓	✗	✗	✓	✗	✗	✗	✗	✗
Indus	✗	✗	✓	✗	✗	✓	✓	✗	✗	✗	✗
bcslt	✗	✗	✓	✓	✗	✗	✗	✗	✓	✗	✓

Table S2: Overview of available PDRMIP data (variables delivered per model).

Variables	CanESM2	MPI-ESM	NorESM1	CESM1-CAM4	CESM1-CAM5	MIROC-SPRINTARS	HadGEM2	HadGEM3	GISS-E2-R	IPSL-CM5A-LR	ECHAM-HAM
**Amon 2D**											
tas	✓	✓	✓	✓	✓	✓	✓	✓	✓	✓	✓
psl	✓	✓	✓	✓	✓	✓	✓	✓	✓	✓	✓
ps	✓	✓	✓	✓	✓	✓	✓	✓	✓	✓	✓
uas	✓	✓	✓	✓	✓	✓	✓	✓	✓	✓	✓
vas	✓	✓	✓	✓	✓	✓	✓	✓	✓	✓	✓
sfcWind	✓	✓	✓	X	✓	✓	✓	✓	✓	X	✓

hurs	✓	X	✓	✓	✓	✓	✓	✓	✓	X	✓
huss	✓	X	✓	✓	✓	✓	✓	✓	✓	X	✓
pr	✓	✓	✓	✓	✓	✓	✓	✓	✓	✓	✓
prsn	✓	✓	✓	✓	X	✓	✓	✓	✓	X	✓
prc	✓	✓	✓	✓	✓	✓	✓	✓	✓	✓	✓
evspsbl	✓	✓	✓	✓	✓	✓	✓	✓	✓	✓	✓
hfls	✓	✓	✓	✓	✓	✓	✓	✓	✓	X	✓
hfss	✓	✓	✓	✓	✓	✓	✓	✓	✓	✓	✓

rlds	✓	✓	✓	✓	✓	✓	✓	✓	✓	X	✓
rlus	✓	✓	✓	✓	✓	✓	✓	✓	✓	X	✓
rsds	✓	✓	✓	✓	✓	✓	✓	✓	✓	X	✓
rsus	✓	✓	✓	✓	✓	✓	✓	✓	✓	X	✓
rsdscs	✓	X	✓	✓	✓	✓	✓	✓	✓	X	X
rsuscs	✓	X	✓	✓	✓	✓	✓	✓	✓	X	X
rldscs	✓	✓	✓	✓	✓	✓	✓	✓	✓	X	✓
rsdt	✓	✓	✓	✓	✓	✓	✓	✓	✓	X	✓

rsut	✓	✓	✓	✓	✓	✓	✓	✓	✓	X	✓
rlut	✓	✓	✓	✓	✓	✓	✓	✓	✓	X	✓
rlutcs	✓	✓	✓	✓	✓	✓	✓	✓	✓	X	✓
rsutcs	✓	✓	✓	✓	✓	✓	✓	✓	✓	X	✓
prw	✓	✓	✓	✓	✓	✓	✓	✓	✓	✓	✓
clt	✓	✓	✓	✓	X	✓	✓	✓	✓	✓	✓
clwvi	✓	✓	✓	✓	X	✓	✓	✓	✓	X	X
clivi	✓	✓	✓	✓	X	✓	✓	✓	✓	X	X

rtmt	✓	✗	✓	✓	✓	✓	✓	✗	✓	✗	✗
ci	✓	✗	✓	✓	✗	✓	✗	✗	✓	✗	✓
sci	✓	✗	✓	✓	✗	✗	✓	✗	✓	✗	✗
**Amon 3D**											
cl	✓	✓	✓	✓	✓	✓	✓	✓	✓	✓	✓
clw	✓	✓	✓	✓	✓	✓	✓	✓	✓	✓	✓
cli	✓	✓	✓	✓	✓	✓	✓	✓	✓	✓	✓
ta	✓	✓	✓	✓	✓	✓	✓	✓	✓	✓	✓
hus	✓	✓	✓	✓	✗	✗	✓	✓	✓	✓	✓

hur	✓	✓	✓	✓	✓	✓	✓	✓	✓	✓	✓
wap	✓	✓	✓	✓	✓	✓	✓	✓	✓	✓	✓
zg	✓	✓	✓	✓	✓	✓	✓	✓	✓	X	✓
ua	✓	✓	✓	✓	✓	✓	✓	✓	✓	✓	✓
va	✓	✓	✓	✓	✓	✓	✓	✓	✓	✓	✓

Table S3: Global and annual multi-model mean top of the atmosphere and surface fluxes for core PDRMIP simulations (variable names described in Tables S5-7).

Variables	Base	co2x2	ch4x3	solar	bcx10	sulx5
			**fsst**			
rsdt	341.05 (10)	-0.00 (10)	0.00 (9)	6.81 (9)	0.00 (9)	0.00 (9)
rsut	101.80 (10)	-0.73 (10)	-0.30 (9)	2.00 (9)	-1.99 (9)	4.13 (9)
rsds	188.60 (10)	0.22 (10)	-0.12 (9)	3.39 (9)	-4.42 (9)	-4.40 (9)
rsus	24.05 (10)	-0.19 (10)	-0.09 (9)	0.28 (9)	-0.71 (9)	-0.33 (9)
rlut	237.51 (10)	-3.10 (10)	-0.83 (9)	0.62 (9)	0.98 (9)	-0.65 (9)
rlds	341.57 (10)	2.34 (10)	0.80 (9)	1.01 (9)	1.28 (9)	-0.22 (9)
rlus	398.14 (10)	1.16 (10)	0.28 (9)	0.62 (9)	0.21 (9)	-0.54 (9)
hfls	86.77 (10)	-2.17 (10)	-0.48 (9)	-0.67 (9)	-2.40 (9)	-0.08 (9)
hfss	19.98 (11)	-0.06 (11)	-0.15 (10)	0.02 (10)	-1.26 (10)	-0.22 (10)
			coupled			
rsdt	341.05 (10)	0.00 (10)	0.00 (10)	6.81 (9)	0.00 (9)	-0.00 (8)
rsut	101.27 (10)	-2.05 (10)	-0.61 (10)	0.76 (9)	-2.30 (9)	6.24 (8)

rsds	189.07 (10)	-1.63 (10)	-0.58 (10)	1.30 (9)	-5.45 (9)	-3.10 (8)
rsus	24.05 (10)	-1.53 (10)	-0.45 (10)	-1.04 (9)	-1.07 (9)	1.36 (8)
rlut	238.50 (10)	1.17 (10)	0.40 (10)	5.15 (9)	2.15 (9)	-5.32 (8)
rlds	343.36 (10)	16.55 (10)	4.49 (10)	15.84 (9)	5.09 (9)	-13.30 (8)
rlus	400.33 (10)	13.11 (10)	3.43 (10)	12.95 (9)	3.33 (9)	-11.93 (8)
hfls	87.42 (10)	3.09 (10)	1.01 (10)	4.93 (9)	-1.41 (9)	-5.36 (8)
hfss	19.93 (11)	-0.57 (11)	-0.27 (11)	-0.51 (10)	-1.32 (10)	0.32 (9)

Table S4: Global and annual multi-model mean meteorological variables for core PDRMIP simulations (variable names described in Table S8-11).

Variables	Base	co2x2	ch4x3	solar	bcx10	sulx5
			**fsst**			
tas	287.54 (11)	0.27 (11)	0.07 (10)	0.13 (10)	0.08 (10)	-0.10 (10)
ts	288.17 (4)	0.26 (4)	0.05 (4)	0.11 (4)	0.05 (3)	-0.08 (3)
pr	3.449E-05 (11)	-0.087E-05 (11)	-0.018E-05 (10)	-0.025E-05 (10)	-0.092E-05 (10)	-0.003E-05 (10)
prc	2.088E-05 (11)	-0.065E-05 (11)	-0.012E-05 (10)	-0.028E-05 (10)	-0.077E-05 (10)	0.002E-05 (10)
huss	0.009545 (9)	-0.000947 (9)	0.000039 (8)	0.000086 (8)	0.000123 (9)	-0.001075 (9)
hurs	76.27 (9)	-2.66 (9)	-8.48 (8)	-8.42 (8)	-7.22 (9)	-10.00 (9)
prw	24.52 (11)	0.21 (11)	0.09 (10)	0.29 (10)	0.43 (10)	-0.12 (10)
evspsbl	2.82E-05 (11)	-0.07E-05 (11)	-0.02E-05 (10)	-0.03E-05 (10)	-0.06E-05 (10)	-0.00E-05 (10)
uas	-0.4671 (11)	-0.0011 (11)	-0.0135 (10)	-0.0158 (10)	-0.0052 (10)	0.0143 (10)
vas	0.1444 (11)	-0.0004 (11)	-0.0017 (10)	0.0046 (10)	0.0181 (10)	-0.0131 (10)
clt	54.80 (10)	-0.24 (10)	-0.13 (9)	-0.02 (9)	-0.14 (9)	0.19 (9)

ps	98534 (11)	-1 (11)	0 (10)	1 (10)	2 (10)	0 (10)
			**coupled**			
tas	287.94 (11)	2.51 (11)	0.69 (11)	2.46 (10)	0.67 (10)	-2.26 (9)
ts	289.54 (4)	2.85 (4)	0.81 (4)	2.80 (4)	0.41 (3)	-3.06 (3)
pr	3.474E-05 (11)	0.126E-05 (11)	0.046E-05 (11)	0.207E-05 (10)	-0.051E-05 (10)	-0.215E-05 (9)
prc	2.120E-05 (11)	0.081E-05 (11)	0.031E-05 (11)	0.116E-05 (10)	-0.056E-05 (10)	-0.147E-05 (9)
huss	0.009737 (9)	0.000275 (9)	0.000368 (9)	0.00156 (8)	0.000473 (9)	-0.001189 (8)
hurs	76.00 (9)	-2.89 (9)	-0.04 (9)	0.00 (8)	0.37 (9)	0.14 (8)
prw	24.90 (11)	4.46 (11)	1.23 (11)	4.94 (10)	1.55 (10)	-3.38 (9)
evspsbl	2.844E-05 (11)	0.098E-05 (11)	0.042E-05 (11)	0.206E-05 (10)	-0.025E-05 (10)	-0.214E-05 (9)
uas	-0.4191 (11)	0.0278 (11)	0.0069 (11)	0.0162 (10)	0.0090 (10)	-0.0068 (9)
vas	0.1042 (11)	0.0060 (11)	-0.0011 (11)	-0.0026 (10)	0.0476 (10)	-0.0622 (9)
clt	54.33 (10)	-0.47 (10)	-0.19 (10)	-0.26 (9)	-0.11 (9)	0.65 (8)
ps	98537 (11)	21 (11)	6 (11)	29 (10)	8 (10)	-23 (9)

Table S5: Global and annual mean short wave (SW) radiative fluxes (rsdt – downward SW radiative fluxes at top of the atmosphere , rsut - upward SW radiative fluxes at top of the atmosphere, rsds – downward SW radiative fluxes at surface, rsus - upward SW radiative fluxes at surface) *(no separate upward and downward fluxes for IPSL-CM5A-LR available).

Model	Base	co2x2	ch4x3	solar	bcx10	sulx5
**FSST:**			**rsdt**			
CanESM2	341.3	341.3	341.3	348.1	341.3	341.3
ECHAM-HAM	340.4	340.4	NA	NA	340.4	340.4
GISS-E2-R	341.6	341.6	341.6	348.4	341.6	341.6
HadGEM2	341.4	341.4	341.4	348.2	341.4	341.4
HadGEM3	341.4	341.4	341.4	348.2	341.4	341.4
MIROC-SPRINTARS	341.7	341.7	341.7	348.6	341.7	341.7
MPI-ESM	340.6	340.6	340.6	347.4	NA	NA
NCAR-CESM1-CAM4	341.4	341.4	341.4	348.1	341.4	341.4
NCAR-CESM1-CAM5	340.4	340.4	340.4	347.2	340.4	340.4
NorESM1	340.3	340.3	340.3	347.1	340.3	340.3
			**rsut**			

CanESM2	100.4	99.7	99.9	102.5	96.8	104.2
ECHAM-HAM	102.0	101.3	NA	NA	99.6	104.9
GISS-E2-R	100.6	100.2	100.4	102.5	98.8	103.7
HadGEM2	97.4	97.0	97.2	99.4	95.4	101.9
HadGEM3	100.8	99.7	100.3	102.5	99.5	110.3
MIROC-SPRINTARS	106.2	105.5	106.1	108.2	105.1	109.6
MPI-ESM	100.8	99.9	100.6	103.0	NA	NA
NCAR-CESM1-CAM4	102.2	101.3	101.9	104.2	100.3	104.5
NCAR-CESM1-CAM5	101.6	100.7	101.4	103.5	100.3	105.4
NorESM1	106.2	105.6	105.9	108.3	103.6	110.1
			**rsds**			
CanESM2	191.9	191.9	190.9	195.1	183.6	188.1
ECHAM-HAM	186.6	186.9	NA	NA	178.7	183.8
GISS-E2-R	193.5	193.6	193.6	197.3	189.5	190.1

HadGEM2	196.4	196 .0	196.4	199.7	193.2	191.4
HadGEM3	195.1	195.6	195.6	199.0	193.2	184.6
MIROC-SPRINTARS	186.2	186.5	185.8	189.8	183.3	182.8
MPI-ESM	185.4	185.8	184.9	188.4	NA	NA
NCAR-CESM1-CAM4	185.2	185.7	185.5	188.4	180.7	182.9
NCAR-CESM1-CAM5	183.4	183.5	182.9	186.6	180.6	179.0
NorESM1	181.6	181.8	181.8	184.8	177.4	177.7
			**rsus**			
CanESM2	27.29	27.13	27.05	27.57	25.80	26.91
ECHAM-HAM	24.79	24.68	NA	NA	23.76	24.58
GISS-E2-R	22.66	22.44	22.55	22.95	22.10	22.41
HadGEM2	24.28	24.10	24.24	24.57	23.71	23.83
HadGEM3	25.16	25.01	25.12	25.52	24.79	24.30
MIROC-SPRINTARS	23.69	23.53	23.62	24.07	23.34	23.50

MPI-ESM	22.91	22.83	22.81	23.17	NA	NA
NCAR-CESM1-CAM4	22.72	22.50	22.72	22.95	22.13	22.57
NCAR-CESM1-CAM5	23.58	23.23	23.44	23.77	22.87	23.27
NorESM1	22.99	22.73	22.89	23.20	22.29	22.80
**Coupled:**			**rsdt**			
CanESM2	341.3	341.3	341.3	348.1	341.3	341.3
ECHAM-HAM	340.4	340.4	340.4	NA	340.4	NA
GISS-E2-R	341.6	341.6	341.6	348.4	341.6	341.6
HadGEM2	341.4	341.4	341.4	348.2	341.4	341.4
HadGEM3	341.4	341.4	341.4	348.2	341.4	341.4
MIROC-SPRINTARS	341.7	341.7	341.7	348.6	341.7	341.7
MPI-ESM	340.6	340.6	340.6	347.4	NA	NA
NCAR-CESM1-CAM4	341.4	341.4	341.4	348.1	341.4	341.4

NCAR-CESM1-CAM5	340.4	340.4	340.4	347.2	340.4	340.4
NorESM1	340.3	340.3	340.3	347.1	340.3	340.3
			**rsut**			
CanESM2	100.8	98.8	100.1	101.4	96.7	106.8
ECHAM-HAM	101.6	99.1	101.4	NA	99.3	NA
GISS-E2-R	100.7	101.1	100.7	103.6	99.0	103.2
HadGEM2	97.9	95.5	97.2	98.9	93.8	104.4
HadGEM3	97.3	91.4	95.3	93.0	95.1	116.7
MIROC-SPRINTARS	107.4	106.4	107.1	109.2	106.4	111.0
MPI-ESM	100.5	99.9	100.4	102.9	NA	NA
NCAR-CESM1-CAM4	101.2	98.6	100.3	101.9	99.4	104.1
NCAR-CESM1-CAM5	99.4	96.9	98.8	99.6	98.2	104.1
NorESM1	105.8	104.3	105.3	107.2	103.4	110.2
**rsds**						

CanESM2	191.9	189.4	191.1	192.2	182.9	189.9
ECHAM-HAM	187.0	185.1	185.9	NA	178.2	NA
GISS-E2-R	192.9	191.0	192.6	194.5	188.5	191.2
HadGEM2	196.3	194.3	195.7	196.8	187.6	194.0
HadGEM3	196.4	197.1	196.8	200.7	194.6	185.7
MIROC-SPRINTARS	187.4	185.8	186.6	188.8	184.2	185.7
MPI-ESM	186.0	183.4	184.8	186.1	NA	NA
NCAR-CESM1-CAM4	184.0	182.6	183.5	185.1	179.1	183.1
NCAR-CESM1-CAM5	184.5	183.1	183.8	186.0	181.6	180.9
NorESM1	184.3	182.7	183.8	185.4	179.1	182.5
			**rsus**			
CanESM2	27.66	25.66	27.10	26.00	25.54	29.64
ECHAM-HAM	24.78	23.13	24.41	NA	23.63	NA
GISS-E2-R	22.32	21.65	22.16	22.14	21.73	22.58

HadGEM2	24.72	22.87	24.19	23.52	22.67	26.78
HadGEM3	24.66	23.03	24.07	23.31	24.02	27.99
MIROC-SPRINTARS	23.81	22.86	23.59	23.32	23.29	24.55
MPI-ESM	23.00	21.90	22.64	22.33	NA	NA
NCAR-CESM1-CAM4	22.14	20.12	21.40	20.70	21.40	22.92
NCAR-CESM1-CAM5	23.14	21.52	22.72	22.03	22.43	23.58
NorESM1	24.35	22.58	23.75	23.11	23.21	25.62

Table S6: Global and annual mean model long wave (LW) radiative fluxes (rlut – upward LW radiative fluxes at top of the atmosphere, rlds – downward LW radiative fluxes at surface, rlus - upward LW radiative fluxes at surface) *(no separate upward and downward fluxes for IPSL-CM5A-LR available).

Model	Base	co2x2	ch4x3	solar	bcx10	sulx5
**FSST:**			**rlut**			
CanESM2	240.2	237.4	239.4	240.9	242.3	239.7
ECHAM-HAM	238.0	234.1	NA	NA	239.1	237.4
GISS-E2-R	240.3	236.7	239.1	240.8	240.9	240.0
HadGEM2	242.9	239.9	242.1	243.5	243.7	242.4
HadGEM3	238.5	236.1	237.7	239.3	239.1	237.4
MIROC-SPRINTARS	235.6	232.7	235.0	236.3	236.1	235.0
MPI-ESM	238.9	235.6	238.2	239.4	NA	NA
NCAR-CESM1-CAM4	233.1	230.4	232.2	233.8	234.2	232.9
NCAR-CESM1-CAM5	233.6	230.4	233.0	234.3	234.5	231.9
NorESM1	234.1	231.2	233.2	234.8	235.3	234.0
			**rlds**			

CanESM2	331.3	333.4	332.6	332.7	334.3	330.7
ECHAM-HAM	348.6	351.2	NA	NA	350.4	348 .0
GISS-E2-R	348.9	351.1	349.6	349.7	349.9	348.7
HadGEM2	333.3	335.4	333.9	334.3	334.3	332.9
HadGEM3	337.6	339.6	338.2	338.4	338.2	338.2
MIROC-SPRINTARS	345.5	347.8	346.1	346.3	346.1	345.2
MPI-ESM	346.0	348.4	346.7	347.0	NA	NA
NCAR-CESM1-CAM4	339.0	341.3	339.8	339.9	340.2	338.3
NCAR-CESM1-CAM5	343.7	346.5	344.3	344.7	344.6	344.5
NorESM1	342.3	344.6	343.3	343.5	343.7	341.4
			**rlus**			
CanESM2	391.4	392.4	391.8	392.1	391.9	390.5
ECHAM-HAM	400.2	401.2	NA	NA	400.1	399.6
GISS-E2-R	405.5	406.6	405.8	405.9	405.7	405.1

HadGEM2	395.7	396.8	395.8	396.3	395.9	395.0
HadGEM3	397.2	398.6	397.6	397.9	397.4	396.6
MIROC-SPRINTARS	398.5	399.6	398.6	399.0	398.5	398.0
MPI-ESM	399.4	400.5	399.6	399.9	NA	NA
NCAR-CESM1-CAM4	397.5	398.7	397.8	398.0	397.6	396.9
NCAR-CESM1-CAM5	396.0	397.6	396.2	396.7	396.5	396.0
NorESM1	400.6	401.6	401.0	401.4	400.9	400.0
**Coupled:**			**rlut**			
CanESM2	240.4	241.4	240.8	245.6	244.2	235.3
ECHAM-HAM	238.5	240.5	238.5	NA	240.7	NA
GISS-E2-R	240.4	238.9	239.9	243.4	241.8	238.5
HadGEM2	243.1	244.4	243.5	248.0	246.8	237.7
HadGEM3	243.3	247.8	244.9	252.9	245.4	226.6
MIROC-SPRINTARS	233.3	233.1	233.4	237.2	234.2	230.3

MPI-ESM	239.2	238.8	239.1	242.8	NA	NA
NCAR-CESM1-CAM4	238.0	240.7	238.9	244.1	239.8	235.1
NCAR-CESM1-CAM5	237.0	238.4	237.3	242.6	238.1	232.7
NorESM1	232.1	232.7	232.3	236.6	234.3	228.7
**rlds**						
CanESM2	331.3	348.8	335.3	350.2	341.0	316.3
ECHAM-HAM	349.5	368.3	353.2	NA	353.9	NA
GISS-E2-R	350.2	361.2	353.3	360.1	353.7	343.9
HadGEM2	333.2	350.6	338.5	349.8	345.4	317.9
HadGEM3	350.0	374.2	357.8	377.5	354.7	312.5
MIROC-SPRINTARS	337.3	348.2	339.8	347.3	338.9	330.3
MPI-ESM	344.5	359.5	348.2	357.8	NA	NA
NCAR-CESM1-CAM4	352.6	373.4	359.9	371.2	356.3	343.4

NCAR-CESM1-CAM5	349.8	366.3	353.4	364.8	351.2	344.0
NorESM1	334.9	348.3	339.4	347.8	339.6	324.6
			**rlus**			
CanESM2	391.3	405.6	394.5	407.2	398.1	377.5
ECHAM-HAM	401.5	416.5	404.0	NA	403.5	NA
GISS-E2-R	406.4	414.4	408.6	413.8	408.5	401.3
HadGEM2	395.7	409.6	399.8	409.2	404.2	381.9
HadGEM3	408.6	428.9	415.0	431.9	412.3	374.0
MIROC-SPRINTARS	394.0	401.9	395.6	401.7	394.7	387.9
MPI-ESM	398.6	409.8	401.2	409.1	NA	NA
NCAR-CESM1-CAM4	409.5	426.5	415.2	424.9	411.7	401.8
NCAR-CESM1-CAM5	402.6	415.8	405.4	415.0	403.4	396.8
NorESM1	394.9	405.4	398.3	405.5	398.1	386.5

Table S7: Global and annual mean surface fluxes of sensible heat (hfss) and latent heat (hfls) *(for IPSL-CM5A-LR data available only for hfss).

Model	Base	co2x2	ch4x3	solar	bcx10	sulx5
**FSST:**			**hfss**			
CanESM2	24.53	24.04	24.10	24.24	22.20	24.42
ECHAM-HAM	23.39	22.89	NA	NA	21.13	23.40
GISS-E2-R	19.29	19.63	19.09	19.32	17.89	18.93
HadGEM2	18.37	18.25	18.24	18.37	17.70	18.27
HadGEM3	18.65	18.89	18.60	18.78	18.14	18.22
IPSL-CM5A-LR	23.11	23.15	23.06	23.26	21.94	22.69
MIROC-SPRINTARS	14.77	14.74	14.64	14.85	13.97	14.59
MPI-ESM	24.70	24.26	24.46	24.55	NA	NA
NCAR-CESM1-CAM4	17.55	17.77	17.49	17.64	16.22	17.29
NCAR-CESM1-CAM5	17.26	17.59	17.11	17.25	16.59	17.13
NorESM1	18.46	18.25	18.39	18.60	17.04	18.26

			**hfls**			
CanESM2	79.24	77.53	78.46	78.97	75.63	79.47
ECHAM-HAM	86.32	84.07	NA	NA	82.29	85.96
GISS-E2-R	93.69	90.76	93.10	93.02	91.19	93.90
HadGEM2	89.37	87.00	89.09	88.58	86.99	89.25
HadGEM3	88.71	86.16	88.23	87.96	87.42	89.06
MIROC-SPRINTARS	94.49	92.61	93.90	93.76	92.67	94.42
MPI-ESM	83.30	81.45	82.76	82.60	NA	NA
NCAR-CESM1-CAM4	81.69	79.69	81.37	80.96	79.39	81.75
NCAR-CESM1-CAM5	87.41	84.86	86.90	86.70	86.14	86.40
NorESM1	83.12	81.53	82.92	82.40	80.76	83.13
**Coupled:**			**hfss**			
CanESM2	24.56	23.16	24.16	23.34	22.22	25.40
ECHAM-HAM	23.47	22.70	23.21	NA	21.40	NA

GISS-E2-R	18.88	18.73	18.54	18.47	17.50	18.92
HadGEM2	18.52	17.90	18.22	17.90	17.00	19.14
HadGEM3	18.16	17.81	17.86	17.86	17.59	19.17
IPSL-CM5A-LR	23.25	22.68	22.93	22.74	22.15	23.34
MIROC-SPRINTARS	14.36	14.04	14.18	14.22	13.58	14.45
MPI-ESM	24.81	23.89	24.53	24.24	NA	NA
NCAR-CESM1-CAM4	17.46	17.01	17.15	16.96	16.13	17.53
NCAR-CESM1-CAM5	17.08	16.68	16.87	16.41	16.40	17.19
NorESM1	18.69	18.33	18.53	18.48	17.28	18.71
			**hfls**			
CanESM2	79.43	82.73	80.22	84.70	77.53	74.22
ECHAM-HAM	86.48	90.27	86.78	NA	83.22	NA
GISS-E2-R	94.44	95.38	95.02	98.07	93.13	91.72
HadGEM2	89.32	92.36	90.57	93.90	87.63	83.73

HadGEM3	93.45	98.65	95.76	102.10	93.71	77.85
MIROC-SPRINTARS	91.38	92.91	91.67	94.51	90.18	88.58
MPI-ESM	82.94	85.37	83.44	86.52	NA	NA
NCAR-CESM1-CAM4	86.85	91.81	88.97	93.20	85.45	83.49
NCAR-CESM1-CAM5	89.98	93.11	90.62	95.14	88.94	86.34
NorESM1	80.07	82.67	81.18	84.14	78.88	76.13

Table S8: Global and annual mean temperature (tas), precipitation (pr) and evaporation (evspsbl).

Model	Base	co2x2	ch4x3	Solar	bcx10	sulx5
**FSST:**			**tas**			
CanESM2	286.8	287.1	286.9	287.0	287.0	286.7
ECHAM-HAM	287.9	288.1	NA	NA	287.9	287.7
GISS-E2-R	289.0	289.3	289.1	289.1	289.1	289.0
HadGEM2	286.8	287.0	286.8	286.9	286.9	286.7
HadGEM3	287.3	287.6	287.4	287.4	287.4	287.2
IPSL-CM5A-LR	287.2	287.5	287.3	287.3	287.2	287.1
MIROC-SPRINTARS	288.1	288.3	288.1	288.2	288.1	288.0
MPI-ESM	288.0	288.3	288.0	288.1	NA	NA
NCAR-CESM1-CAM4	287.3	287.6	287.3	287.4	287.4	287.2
NCAR-CESM1-CAM5	286.9	287.2	286.9	287.0	287.0	286.9
NorESM1	287.8	288.0	287.8	287.9	287.9	287.6

			**pr**			
CanESM2	3.162E-05	3.094E-05	3.131E-05	3.151E-05	3.018E-05	3.171E-05
ECHAM-HAM	3.459E-05	3.369E-05	NA	NA	3.297E-05	3.444E-05
GISS-E2-R	3.747E-05	3.630E-05	3.724E-05	3.720E-05	3.648E-05	3.756E-05
HadGEM2	3.573E-05	3.477E-05	3.561E-05	3.541E-05	3.478E-05	3.568E-05
HadGEM3	3.550E-05	3.448E-05	3.531E-05	3.520E-05	3.498E-05	3.565E-05
IPSL-CM5A-LR	3.234E-05	3.140E-05	3.229E-05	3.223E-05	3.178E-05	3.229E-05
MIROC-SPRINTARS	3.780E-05	3.704E-05	3.756E-05	3.751E-05	3.707E-05	3.777E-05
MPI-ESM	3.336E-05	3.262E-05	3.314E-05	3.308E-05	NA	NA
NCAR-CESM1-CAM4	3.265E-05	3.186E-05	3.252E-05	3.236E-05	3.173E-05	3.268E-05
NCAR-CESM1-CAM5	3.496E-05	3.394E-05	3.476E-05	3.468E-05	3.445E-05	3.456E-05
NorESM1	3.323E-05	3.259E-05	3.314E-05	3.294E-05	3.228E-05	3.323E-05
			**evspsbl**			
CanESM2	3.162E-05	3.094E-05	3.131E-05	3.151E-05	3.019E-05	3.171E-05

ECHAM-HAM	-3.450E-05	-3.360E-05	NA	NA	-3.289E-05	-3.435E-05
Sign convention downward and opposite to the other models						
GISS-E2-R	3.747E-05	3.630E-05	3.724E-05	3.721E-05	3.648E-05	3.756E-05
HadGEM2	3.570E-05	3.475E-05	3.559E-05	3.538E-05	3.475E-05	3.565E-05
HadGEM3	3.532E-05	3.429E-05	3.513E-05	3.502E-05	3.481E-05	3.547E-05
IPSL-CM5A-LR	3.233E-05	3.139E-05	3.228E-05	3.222E-05	3.177E-05	3.228E-05
MIROC-SPRINTARS	3.780E-05	3.705E-05	3.756E-05	3.751E-05	3.707E-05	3.777E-05
MPI-ESM	3.329E-05	3.255E-05	3.308E-05	3.301E-05	NA	NA
NCAR-CESM1-CAM4	3.265E-05	3.186E-05	3.253E-05	3.236E-05	3.174E-05	3.268E-05
NCAR-CESM1-CAM5	3.496E-05	3.394E-05	3.476E-05	3.468E-05	3.445E-05	3.456E-05
NorESM1	3.323E-05	3.259E-05	3.315E-05	3.294E-05	3.228E-05	3.323E-05
**Coupled:**			**tas**			
CanESM2	286.8	289.5	287.4	289.8	288.1	284.1
ECHAM-HAM	288.1	291.1	288.7	NA	288.6	NA

GISS-E2-R	289.2	290.7	289.6	290.6	289.6	288.3
HadGEM2	286.7	289.5	287.5	289.3	288.4	284.0
HadGEM3	289.4	293.1	290.6	293.6	290.1	282.8
IPSL-CM5A-LR	287.4	289.9	288.4	290.3	288.1	285.4
MIROC-SPRINTARS	287.4	288.9	287.7	288.8	287.6	286.2
MPI-ESM	287.8	290.1	288.3	289.8	NA	NA
NCAR-CESM1-CAM4	289.5	292.7	290.6	292.4	290.0	288.0
NCAR-CESM1-CAM5	288.2	290.7	288.7	290.5	288.4	287.1
NorESM1	286.7	288.8	287.4	288.7	287.4	285.0
			**pr**			
CanESM2	3.170E-05	3.301E-05	3.202E-05	3.380E-05	3.094E-05	2.963E-05
ECHAM-HAM	3.463E-05	3.618E-05	3.477E-05	NA	3.333E-05	NA
GISS-E2-R	3.778E-05	3.815E-05	3.801E-05	3.923E-05	3.725E-05	3.669E-05
HadGEM2	3.570E-05	3.692E-05	3.620E-05	3.754E-05	3.504E-05	3.347E-05

HadGEM3	3.741E-05	3.952E-05	3.835E-05	4.091E-05	3.753E-05	3.113E-05
IPSL-CM5A-LR	3.252E-05	3.401E-05	3.352E-05	3.541E-05	3.252E-05	3.036E-05
MIROC-SPRINTARS	3.655E-05	3.716E-05	3.667E-05	3.780E-05	3.607E-05	3.543E-05
MPI-ESM	3.321E-05	3.420E-05	3.341E-05	3.466E-05	NA	NA
NCAR-CESM1-CAM4	3.472E-05	3.670E-05	3.557E-05	3.726E-05	3.416E-05	3.337E-05
NCAR-CESM1-CAM5	3.599E-05	3.724E-05	3.625E-05	3.806E-05	3.557E-05	3.453E-05
NorESM1	3.201E-05	3.305E-05	3.245E-05	3.363E-05	3.153E-05	3.043E-05
**evspsbl**						
CanESM2	3.170E-05	3.301E-05	3.202E-05	3.380E-05	3.095E-05	2.963E-05
ECHAM-HAM	-3.456E-05	-3.608E-05	-3.468E-05	NA	-3.326E-05	NA
Sign convention downward and opposite to the other models						
GISS-E2-R	3.778E-05	3.815E-05	3.801E-05	3.923E-05	3.725E-05	3.669E-05
HadGEM2	3.568E-05	3.690E-05	3.618E-05	3.752E-05	3.501E-05	3.344E-05
HadGEM3	3.721E-05	3.926E-05	3.813E-05	4.064E-05	3.732E-05	3.1E-05

IPSL-CM5A-LR	3.251E-05	3.400E-05	3.352E-05	3.540E-05	3.252E-05	3.035E-05
MIROC-SPRINTARS	3.655E-05	3.717E-05	3.667E-05	3.780E-05	3.607E-05	3.543E-05
MPI-ESM	3.315E-05	3.412E-05	3.335E-05	3.458E-05	NA	NA
NCAR-CESM1-CAM4	3.472E-05	3.670E-05	3.557E-05	3.726E-05	3.416E-05	3.337E-05
NCAR-CESM1-CAM5	3.599E-05	3.724E-05	3.625E-05	3.806E-05	3.558E-05	3.453E-05
NorESM1	3.201E-05	3.305E-05	3.245E-05	3.363E-05	3.153E-05	3.043E-05

Table S9: Global and annual mean surface specific humidity (huss), surface relative humidity (hurs) and surface pressure (ps) *(no data for HadGEM3, IPSL-CM5A-LR or MPI-ESM for huss and hurs or for GISS-E2-R for hurs available).

Model	Base	co2x2	ch4x3	solar	bcx10	sulx5
**FSST:**			**huss**			
CanESM2	0.01082	0.01092	0.01089	0.01093	0.01100	0.01074
ECHAM-HAM	0.009732	0.000480	NA	NA	0.009946	0.000460
GISS-E2-R	0.010336	0.010405	0.010386	0.010435	0.010457	0.010301
HadGEM2	0.009426	0.009501	0.009448	0.009503	0.009510	0.009356
HadGEM3	0.009684	0.009755	0.009714	0.009758	0.009745	0.009615
MIROC-SPRINTARS	0.009552	0.009661	0.009582	0.009623	0.009622	0.009501
NCAR-CESM1-CAM4	0.010341	0.010427	0.010374	0.010423	0.010474	0.010302
NCAR-CESM1-CAM5	0.010131	0.010244	0.010170	0.010226	0.010212	0.010122
NorESM1	0.010468	0.010571	0.010511	0.010553	0.010627	0.010424
			**hurs**			
CanESM2	81.685	81.644	81.820	81.853	82.167	81.737

ECHAM-HAM	75.151	52.931	NA	NA	76.105	53.151
GISS-E2-R	73.810	73.383	5.719	5.723	5.676	5.724
HadGEM2	76.055	75.751	76.050	76.143	76.297	76.029
HadGEM3	76.375	76.007	76.339	76.462	76.573	76.243
MIROC-SPRINTARS	77.667	77.656	77.721	77.773	77.918	77.708
NCAR-CESM1-CAM4	79.324	79.058	79.309	79.431	79.784	79.437
NCAR-CESM1-CAM5	78.803	78.470	78.863	78.897	78.935	78.719
NorESM1	78.252	78.289	78.274	78.297	78.728	78.378
			**ps**			
CanESM2	9846E+04	9.846E+04	9.846E+04	9.846E+04	9.846E+04	9.846E+04
ECHAM-HAM	9.855E+04	9.855E+04	NA	NA	9.855E+04	9.855E+04
GISS-E2-R	9.840E+04	9.840E+04	9.840E+04	9.840E+04	9.840E+04	9.840E+04
HadGEM2	9.857E+04	9.855E+04	9.857E+04	9.858E+04	9.858E+04	9.857E+04
HadGEM3	9.852E+04	9.852E+04	9.852E+04	9.852E+04	9.852E+04	9.852E+04

IPSL-CM5A-LR	9.851E+04	9.851E+04	9.851E+04	9.851E+04	9.851E+04	9.851E+04
MIROC-SPRINTARS	9.868E+04	9.868E+04	9.868E+04	9.868E+04	9.868E+04	9.868E+04
MPI-ESM	9.855E+04	9.855E+04	9.855E+04	9.855E+04	NA	NA
NCAR-CESM1-CAM4	9.854E+04	9.854E+04	9.854E+04	9.854E+04	9.854E+04	9.854E+04
NCAR-CESM1-CAM5	9.854E+04	9.854E+04	9.854E+04	9.854E+04	9.854E+04	9.854E+04
NorESM1	9.855E+04	9.855E+04	9.855E+04	9.855E+04	9.855E+04	9.854E+04
**Coupled:**			**huss**			
CanESM2	0.010825	0.012484	0.011192	0.012760	0.011710	0.009417
ECHAM-HAM	0.009788	0.000661	0.000508	NA	0.010218	NA
GISS-E2-R	0.010476	0.011392	0.010769	0.011469	0.010861	0.009867
HadGEM2	0.009401	0.010842	0.009831	0.010848	0.010437	0.008123
HadGEM3	0.010861	0.013169	0.011587	0.013640	0.011321	0.007459
MIROC-SPRINTARS	0.008850	0.009690	0.009035	0.009716	0.008993	0.008286

NCAR-CESM1-CAM4	0.011606	0.013506	0.012248	0.013439	0.011976	0.010774
NCAR-CESM1-CAM5	0.010653	0.012136	0.010961	0.012141	0.010782	0.010038
NorESM1	0.009821	0.010879	0.010155	0.010944	0.010243	0.009023
			**hurs**			
CanESM2	81.685	81.541	81.704	81.668	81.936	81.721
ECHAM-HAM	74.789	51.161	52.358	NA	75.655	NA
GISS-E2-R	74.017	73.651	74.103	74.286	74.435	73.946
HadGEM2	75.987	75.633	75.983	75.999	76.555	76.145
HadGEM3	75.899	75.380	75.856	75.701	76.051	76.180
MIROC-SPRINTARS	77.490	77.538	77.563	77.627	77.708	77.605
NCAR-CESM1-CAM4	78.692	78.376	78.645	78.655	79.063	78.831
NCAR-CESM1-CAM5	78.150	77.745	78.167	78.168	78.319	78.300
NorESM1	78.304	78.014	78.231	78.145	78.630	78.604

			**ps**			
CanESM2	9.846E+04	9.846E+04	9.846E+04	9.846E+04	9.846E+04	9.846E+04
ECHAM-HAM	9.855E+04	9.855E+04	9.855E+04	NA	9.855E+04	NA
GISS-E2-R	9.840E+04	9.840E+04	9.840E+04	9.840E+04	9.840E+04	9.840E+04
HadGEM2	9.857E+04	9.859E+04	9.858E+04	9.862E+04	9.861E+04	9.853E+04
HadGEM3	9.855E+04	9.862E+04	9.857E+04	9.863E+04	9.857E+04	9.847E+04
IPSL-CM5A-LR	9.851E+04	9.851E+04	9.851E+04	9.851E+04	9.851E+04	9.851E+04
MIROC-SPRINTARS	9.865E+04	9.868E+04	9.866E+04	9.868E+04	9.866E+04	9.864E+04
MPI-ESM	9.855E+04	9.855E+04	9.855E+04	9.855E+04	NA	NA
NCAR-CESM1-CAM4	9.858E+04	9.863E+04	9.860E+04	9.864E+04	9.859E+04	9.855E+04
NCAR-CESM1-CAM5	9.855E+04	9.860E+04	9.856E+04	9.860E+04	9.856E+04	9.854E+04
NorESM1	9.852E+04	9.856E+04	9.853E+04	9.856E+04	9.854E+04	9.850E+04

Table S10: Global and annual mean convective precipitation (prc), total water vapour path (prw) and total clouds fraction (clt) *(no data available for clt for NCAR-CESM1-CAM5).

Model	Base	co2x2	ch4x3	solar	bcx10	sulx5
**FSST:**			**prc**			
CanESM2	1.714E-05	1.643E-05	1.686E-05	1.671E-05	1.556E-05	1.713E-05
ECHAM-HAM	2.311E-05	2.255E-05	NA	NA	2.174E-05	2.315E-05
GISS-E2-R	1.541E-05	1.464E-05	1.526E-05	1.534E-05	1.498E-05	1.546E-05
HadGEM2	2.659E-05	2.583E-05	2.648E-05	2.626E-05	2.574E-05	2.666E-05
HadGEM3	2.586E-05	2.511E-05	2.573E-05	2.556E-05	2.539E-05	2.626E-05
IPSL-CM5A-LR	1.577E-05	1.508E-05	1.581E-05	1.552E-05	1.514E-05	1.571E-05
MIROC-SPRINTARS	1.918E-05	1.859E-05	1.906E-05	1.893E-05	1.864E-05	1.921E-05
MPI-ESM	2.144E-05	2.103E-05	2.129E-05	2.114E-05	NA	NA
NCAR-CESM1-CAM4	2.000E-05	1.941E-05	1.996E-05	1.973E-05	1.928E-05	2.009E-05
NCAR-CESM1-CAM5	2.478E-05	2.387E-05	2.458E-05	2.446E-05	2.438E-05	2.428E-05
NorESM1	2.025E-05	1.982E-05	2.022E-05	1.997E-05	1.951E-05	2.029E-05

			**prw**			
CanESM2	22.03	22.21	22.24	22.42	23.00	21.93
ECHAM-HAM	26.48	26.72	NA	NA	27.18	26.17
GISS-E2-R	24.88	24.98	24.92	25.14	25.21	24.81
HadGEM2	22.17	22.34	22.22	22.45	22.58	21.96
HadGEM3	23.32	23.44	23.36	23.53	23.49	23.25
IPSL-CM5A-LR	23.81	24.09	23.88	24.10	24.18	23.72
MIROC-SPRINTARS	25.12	25.38	25.23	25.36	25.31	24.95
MPI-ESM	24.82	25.01	24.93	25.15	NA	NA
NCAR-CESM1-CAM4	25.52	25.74	25.58	25.79	25.96	25.36
NCAR-CESM1-CAM5	25.56	25.84	25.67	25.87	25.81	25.72
NorESM1	26.03	26.26	26.13	26.34	26.54	25.88
			**clt**			
CanESM2	61.37	61.02	61.33	61.44	61.29	61.45

ECHAM-HAM	68.11	68.08	NA	NA	67.95	68.79
GISS-E2-R	61.18	60.98	61.20	60.94	60.79	61.33
HadGEM2	53.41	53.00	53.14	53.37	53.21	53.74
HadGEM3	64.46	63.84	64.08	64.18	64.25	65.41
IPSL-CM5A-LR	0.5633	0.5555	0.5576	0.5611	0.5487	0.5603
(Given as fraction and instead of percent for the other models.)						
MIROC-SPRINTARS	67.91	67.48	67.65	67.84	67.70	68.01
MPI-ESM	62.56	62.13	62.46	62.55	NA	NA
NCAR-CESM1-CAM4	53.87	53.85	53.70	53.97	53.86	53.69
NorESM1	54.38	54.53	54.39	54.70	54.43	53.98
**Coupled:**			**prc**			
CanESM2	1.714E-05	1.711E-05	1.716E-05	1.727E-05	1.580E-05	1.627E-05
ECHAM-HAM	2.327E-05	2.457E-05	2.344E-05	NA	2.198E-05	NA
GISS-E2-R	1.565E-05	1.608E-05	1.592E-05	1.685E-05	1.567E-05	1.496E-05

HadGEM2	2.657E-05	2.768E-05	2.699E-05	2.805E-05	2.582E-05	2.476E-05
HadGEM3	2.762E-05	2.975E-05	2.848E-05	3.076E-05	2.770E-05	2.255E-05
IPSL-CM5A-LR	1.581E-05	1.542E-05	1.607E-05	1.579E-05	1.533E-05	1.481E-05
MIROC-SPRINTARS	1.862E-05	1.888E-05	1.867E-05	1.921E-05	1.819E-05	1.797E-05
MPI-ESM	2.144E-05	2.218E-05	2.157E-05	2.232E-05	NA	NA
NCAR-CESM1-CAM4	2.151E-05	2.343E-05	2.234E-05	2.347E-05	2.106E-05	2.049E-05
NCAR-CESM1-CAM5	2.598E-05	2.671E-05	2.613E-05	2.725E-05	2.557E-05	2.469E-05
NorESM1	1.962E-05	2.034E-05	1.991E-05	2.058E-05	1.910E-05	1.880E-05
			**prw**			
CanESM2	22.07	26.43	23.04	27.42	24.77	18.56
ECHAM-HAM	26.62	31.88	27.52	NA	28.09	NA
GISS-E2-R	25.29	28.19	26.15	28.56	26.51	23.35
HadGEM2	22.09	26.31	23.34	26.50	25.68	18.47
HadGEM3	26.83	33.78	28.97	35.51	28.22	17.31

IPSL-CM5A-LR	23.99	29.40	26.12	30.76	25.73	20.60
MIROC-SPRINTARS	22.44	25.00	23.04	25.26	22.86	20.71
MPI-ESM	24.38	28.37	25.31	28.41	NA	NA
NCAR-CESM1-CAM4	29.38	35.09	31.28	35.17	30.63	26.80
NCAR-CESM1-CAM5	26.88	31.39	27.82	31.57	27.27	25.17
NorESM1	23.91	27.04	24.86	27.44	25.23	21.46
			**clt**			
CanESM2	61.38	60.88	61.27	61.12	61.22	61.81
ECHAM-HAM	67.71	66.44	67.54	NA	67.73	NA
GISS-E2-R	61.22	60.97	61.15	60.77	60.73	61.41
HadGEM2	53.48	53.03	53.28	53.33	53.30	54.20
HadGEM3	62.86	60.32	61.93	60.05	62.27	67.96
IPSL-CM5A-LR (Given as fraction and instead of percent for the other models.)	0.5627	0.5407	0.5514	0.5414	0.5453	0.5788

MIROC-SPRINTARS	66.95	66.82	66.70	67.08	66.78	66.99
MPI-ESM	62.00	61.51	61.90	61.90	NA	NA
NCAR-CESM1-CAM4	53.58	53.90	53.66	54.12	53.84	53.13
NorESM1	53.46	54.08	53.57	54.25	53.77	52.61

Table S11: Global and annual mean surface wind (uas – eastward component of wind near surface, vas – northward component of wind near surface) *(no data for HadGEM3).

Model	Base	co2x2	ch4x3	solar	bcx10	sulx5
**FSST:**			**uas**			
CanESM2	-0.3189	-0.3254	-0.3507	-0.3629	-0.3423	-0.2951
ECHAM-HAM	-0.4150	-0.4118	NA	NA	-0.4336	-0.3412
GISS-E2-R	-0.1153	-0.0977	-0.1231	-0.1320	-0.1089	-0.1114
HadGEM2	-0.4256	-0.4474	-0.4497	-0.4458	-0.4450	-0.4167
HadGEM3	-0.5073	-0.5272	-0.5326	-0.5146	-0.5083	-0.4863
IPSL-CM5A-LR	-0.3811	-0.3716	-0.4030	-0.3815	-0.3845	-0.3662
MIROC-SPRINTARS	-0.4229	-0.4173	-0.4316	-0.4454	-0.4338	-0.4279
MPI-ESM	-0.3050	-0.3197	-0.2952	-0.3343	NA	NA
NCAR-CESM1-CAM4	-0.7745	-0.7699	-0.7793	-0.7664	-0.7710	-0.7664
NCAR-CESM1-CAM5	-0.7168	-0.7346	-0.7319	-0.7373	-0.7242	-0.7363
NorESM1	-0.7952	-0.7674	-0.7999	-0.8005	-0.7735	-0.7816

			vas			
CanESM2	0.1294	0.1393	0.1369	0.1400	0.1784	0.1134
ECHAM-HAM	0.2598	0.2421	NA	NA	0.2592	0.2280
GISS-E2-R	0.1016	0.1079	0.1031	0.1066	0.1070	0.0917
HadGEM2	0.1588	0.1666	0.1538	0.1679	0.1871	0.1522
HadGEM3	0.1399	0.1425	0.1342	0.1437	0.1391	0.1247
IPSL-CM5A-LR	0.1046	0.1062	0.1060	0.1125	0.1105	0.0990
MIROC-SPRINTARS	0.1634	0.1568	0.1626	0.1610	0.1754	0.1569
MPI-ESM	0.1452	0.1444	0.1415	0.1535	NA	NA
NCAR-CESM1-CAM4	0.1506	0.1399	0.1463	0.1476	0.1681	0.1319
NCAR-CESM1-CAM5	0.1714	0.1812	0.1695	0.1744	0.1975	0.1626
NorESM1	0.0838	0.0767	0.0784	0.0871	0.1224	0.0722
**Coupled:**			**uas**			
CanESM2	-0.3113	-0.2745	-0.3073	-0.2976	-0.2920	-0.2925

ECHAM-HAM	-0.3878	-0.3259	-0.3684	NA	-0.3941	NA
GISS-E2-R	-0.1214	-0.0831	-0.1173	-0.1163	-0.1188	-0.1221
HadGEM2	-0.4022	-0.3606	-0.3828	-0.3592	-0.3719	-0.3656
HadGEM3	-0.4376	-0.4170	-0.4367	-0.4314	-0.4399	-0.5609
IPSL-CM5A-LR	-0.3940	-0.3552	-0.3811	-0.3570	-0.3721	-0.3812
MIROC-SPRINTARS	-0.4494	-0.4118	-0.4351	-0.4215	-0.4436	-0.4400
MPI-ESM	-0.2620	-0.2623	-0.2750	-0.2633	NA	NA
NCAR-CESM1-CAM4	-0.6235	-0.6113	-0.6209	-0.6066	-0.6247	-0.6470
NCAR-CESM1-CAM5	-0.5013	-0.4951	-0.4873	-0.4918	-0.5093	-0.4963
NorESM1	-0.7308	-0.7191	-0.7214	-0.7270	-0.7019	-0.7270
			**vas**			
CanESM2	0.1296	0.1341	0.1338	0.1417	0.1842	0.0316
ECHAM-HAM	0.2516	0.3114	0.2560	NA	0.3254	NA
GISS-E2-R	0.1000	0.0930	0.0938	0.0963	0.1191	0.0773

HadGEM2	0.1376	0.1675	0.1330	0.1506	0.2639	-0.0309
HadGEM3	0.0242	0.0406	0.0277	0.0386	0.0510	-0.0182
IPSL-CM5A-LR	0.1135	0.0898	0.0982	0.0872	0.1233	0.0809
MIROC-SPRINTARS	0.1373	0.1325	0.1354	0.1389	0.1470	0.0814
MPI-ESM	0.1581	0.1598	0.1595	0.1481	NA	NA
NCAR-CESM1-CAM4	0.0057	-0.0119	-0.0095	-0.0116	0.0641	-0.0557
NCAR-CESM1-CAM5	0.0470	0.0380	0.0520	0.0207	0.0933	-0.0016
NorESM1	0.0462	0.0620	0.0471	0.0620	0.0971	0.0161

Table S12 – phase 2: Global and annual multi-model mean energy fluxes[i].

Variables	Base	cfc12	cfc11	n2o1p	Indus	bcslt
			**fsst**			
rsdt	341.06 (10)	0.00 (7)	-0.00 (4)	0.00 (5)	0.00 (3)	0.00 (4)
rsut	101.81 (10)	-0.33 (7)	-0.31 (4)	-0.29 (5)	-0.08 (3)	-1.15 (4)

rsds	188.60 (10)	0.25 (7)	0.28 (4)	0.156 (5)	0.05 (3)	-3.21 (4)
rsus	24.05 (10)	-0.04 (7)	0.01 (4)	-0.08 (5)	-0.05 (3)	-0.43 (4)
rlut	237.51 (10)	-1.12 (7)	-1.00 (4)	-1.32 (5)	0.12 (3)	0.40 (4)
rlds	341.57 (10)	1.13 (7)	0.95 (4)	1.01 (5)	0.12 (3)	0.69 (4)
rlus	398.14 (10)	0.35 (7)	0.29 (4)	0.40 (5)	0.21 (3)	-0.06 (4)
hfls	86.77 (10)	-0.38 (7)	-0.39 (4)	-0.65 (5)	-0.10 (3)	-1.58 (4)
hfss	19.98 (11)	-0.01 (7)	-0.01 (4)	-0.13 (5)	0.15 (3)	-1.19 (4)
			**coupled**			
rsdt	341.05 (10)	0.00 (8)	-0.00 (3)	-0.00 (5)	-0.00 (3)	0.00 (4)
rsut	101.27 (10)	-0.90 (8)	-0.67 (3)	-0.74 (5)	-0.35 (3)	-0.90 (4)
rsds	189.07 (10)	-0.37 (8)	-0.44 (3)	-0.69 (5)	0.53 (3)	-3.34 (4)
rsus	24.05 (10)	-0.57 (8)	-0.45 (3)	-0.67 (5)	0.16 (3)	-0.51 (4)
rlut	238.50 (10)	0.60 (8)	0.46 (3)	0.46 (5)	0.35 (3)	0.80 (4)
rlds	343.36 (10)	6.26 (8)	5.06 (3)	6.33 (5)	0.80 (3)	2.13 (4)
rlus	400.33 (10)	4.64 (8)	3.75 (3)	4.94 (5)	0.84 (3)	1.17 (4)

hfls	87.42 (10)	1.77 (8)	1.33 (3)	1.48 (5)	0.12 (3)	-0.87 (4)
hfss	19.93 (11)	-0.22 (8)	-0.20 (3)	-0.35 (5)	0.20 (3)	-1.10 (4)

Table S13 phase 2: Global and annual multi-model mean meteorological variables[i].

Variables	Base	cfc12	cfc11	n2op1	Indus	bcslt
			**fsst**			
tas	287.54 (11)	0.07 (7)	0.06 (4)	0.09 (5)	0.04 (3)	0.11 (4)
ts	288.17 (4)	0.07 (2)	0.03 (1)	0.05 (1)	NA (0)	0.02 (1)
pr	3.4491E-05 (11)	-0.0153E-05 (7)	-0.0157E-05 (4)	-0.0261E-05 (5)	-0.0041E-05 (3)	-0.0635E-05 (4)
prc	2.0883E-05 (11)	-0.0086E-05 (7)	-0.0091E-05 (4)	-0.0167E-05 (5)	-0.0039E-05 (3)	-0.0445E-05 (4)
huss	0.0095452 (9)	-0.0012985 (7)	-0.0022938(4)	-0.0018155 (5)	-0.0000012 (3)	0.0001027 (4)
hurs	76.27 (9)	-3.19(7)	-5.52 (4)	-4.44 (5)	-0.08 (3)	0.26 (3)
prw	24.52 (11)	0.09 (7)	0.07 (4)	0.10 (5)	0.00 (3)	0.23 (4)
evspsbl	2.8201E-05 (11)	-0.0075E-05 (7)	-0.0006E-05 (4)	-0.0091E-05 (5)	-0.0039E-05 (3)	-0.0337E-05 (4)
uas	-0.46713 (11)	-0.00954 (7)	-0.00454 (4)	0.01069 (5)	0.01026 (3)	-0.00775 (4)

vas	0.14435 (11)	0.00053 (7)	0.00139 (4)	- 0.00664 (5)	0.00569 (3)	0.00458 (4)
clt	54.803 (10)	-0.283 (6)	-0.248 (4)	-0.129 (5)	-0.016 (3)	0.157 (4)
ps	98534 (11)	1 (7)	1 (4)	1 (5)	0 (3)	2 (4)
			**coupled**			
tas	287.94 (11)	0.88 (8)	0.72 (3)	0.96 (5)	0.16 (3)	0.25 (4)
ts	289.54 (4)	1.17 (3)	NA (0)	1.09 (1)	NA (0)	0.24 (1)
pr	3.4744E-05 (11)	0.0709E-05 (8)	0.0532E-05 (3)	0.0591E-05 (5)	0.0049E-05 (3)	-0.0348E-05 (4)
prc	2.1204E-05 (11)	0.0569E-05 (8)	0.0395E-05 (3)	0.0486E-05 (5)	0.0056E-05 (3)	-0.0262E-05 (4)
huss	0.0097366 (8)	0.0004559 (7)	0.0002657 (3)	0.0004282 (5)	0.0000694 (3)	0.0002348 (4)
hurs	75.999 (7)	-0.093 (6)	-0.159 (3)	-0.168 (5)	-0.090 (3)	0.345 (3)
prw	24.9 (11)	1.5 (7)	1.2 (3)	1.6 (5)	0.2 (3)	0.7 (4)
evspsbl	2.8437E-05 (11)	0.0439E-05 (7)	0.0121E-05 (3)	0.0338E-05 (5)	0.0049E-05 (3)	-0.0001E-05 (4)
uas	-0.41911 (10)	0.00917 (7)	0.01216 (3)	0.01466 (5)	0.01514 (3)	-0.01311 (4)
vas	0.10422 (10)	-0.00137 (7)	0.00588 (3)	0.00304 (5)	0.00652 (2)	0.01160 (4)

clt	54.332 (10)	-0.428 (7)	-0.456 (3)	-0.245 (5)	0.056 (3)	0.304 (4)
ps	98537 (10)	12 (7)	8 (3)	12 (5)	2 (3)	4 (4)

Table S14 phase 2: Global and annual multi-model mean short wave radiation fluxes *(no data for CanESM2, IPSL-CM5A or MPI-ESM available).

Model	Base	cfc12	cfc11	n2o1p	Indus	bcslt
**fsst:**			**rsut**			
ECHAM-HAM	102.02	101.71	101.74	101.98	NA	101.82
GISS-E2-R	100.64	100.56	NA	NA	NA	99.055
HadGEM2	97.352	96.531	96.563	96.356	96.696	NA
HadGEM3	100.84	100.28	NA	NA	NA	NA
MIROC-SPRINTARS	106.21	105.91	105.87	105.96	106.19	NA
NCAR-CESM1-CAM4	102.2	101.76	101.82	101.79	NA	100.93
NCAR-CESM1-CAM5	101.58	100.95	NA	NA	NA	NA
NorESM1	106.17	105.87	NA	105.85	106.04	105

			**rsds**			
ECHAM-HAM	186.65	186.85	186.9	186.53	NA	182.78
GISS-E2-R	193.48	193.41	NA	NA	NA	190.86
HadGEM2	196.41	198.08	198.17	198.27	198.05	NA
HadGEM3	195.11	195.44	NA	NA	NA	NA
MIROC-SPRINTARS	186.21	186.51	186.53	186.32	186.19	NA
NCAR-CESM1-CAM4	185.16	185.61	185.56	185.52	NA	181.94
NCAR-CESM1-CAM5	183.35	183.87	NA	NA	NA	NA
NorESM1	181.63	181.91	NA	181.83	181.79	179.49
			**rsus**			
ECHAM-HAM	24.788	24.755	24.795	24.759	NA	24.294
GISS-E2-R	22.664	22.551	NA	NA	NA	22.309
HadGEM2	24.277	24.956	25.065	24.954	24.968	NA
HadGEM3	25.157	25.091	NA	NA	NA	NA

MIROC-SPRINTARS	23.693	23.717	23.702	23.593	23.654	NA
NCAR-CESM1-CAM4	22.717	22.705	22.732	22.678	NA	22.306
NCAR-CESM1-CAM5	23.575	23.525	NA	NA	NA	NA
NorESM1	22.988	22.977	NA	22.9	22.993	22.527
**Coupled:**			**rsut**			
ECHAM-HAM	101.56	100.79	100.94	100.98	NA	101.76
GISS-E2-R	100.71	100.81	NA	NA	NA	99.246
HadGEM2	97.898	97.464	97.237	96.911	97.19	NA
HadGEM3	97.339	95.12	NA	NA	NA	NA
MIROC-SPRINTARS	107.37	106.86	106.88	106.92	107.36	NA
NCAR-CESM1-CAM4	101.23	99.999	NA	100.37	NA	100.14
NCAR-CESM1-CAM5	99.353	98.207	NA	NA	NA	NA
NorESM1	105.83	105.04	NA	105.25	105.51	104.61

			**rsds**			
ECHAM-HAM	187.01	186.34	186.44	186.11	NA	182.73
GISS-E2-R	192.91	192.4	NA	NA	NA	189.92
HadGEM2	196.33	195.37	195.43	195.03	197.59	NA
HadGEM3	196.44	196.91	NA	NA	NA	NA
MIROC-SPRINTARS	187.42	187.06	187.14	186.87	187.34	NA
NCAR-CESM1-CAM4	184.05	183.5	NA	183.44	NA	180.31
NCAR-CESM1-CAM5	184.5	184.24	NA	NA	NA	NA
NorESM1	184.31	183.74	NA	183.76	184.64	181.96
			**rsus**			
ECHAM-HAM	24.783	24.195	24.299	24.225	NA	24.384
GISS-E2-R	22.317	22.132	NA	NA	NA	21.869
HadGEM2	24.725	24.138	24.021	23.681	25.286	NA
HadGEM3	24.656	24.082	NA	NA	NA	NA

MIROC-SPRINTARS	23.806	23.519	23.549	23.391	23.748	NA
NCAR-CESM1-CAM4	22.14	21.216	NA	21.382	NA	21.646
NCAR-CESM1-CAM5	23.14	22.426	NA	NA	NA	NA
NorESM1	24.354	23.559	NA	23.696	24.312	23.654

Table S15 phase 2: Global and annual multi-model mean long wave radiation fluxes *(no data for CanESM2, IPSL-CM5A or MPI-ESM available).

Model	Base	cfc12	cfc11	n2o1p	Indus	bcslt
**FSST:**			**rlut**			
ECHAM-HAM	237.97	236.52	236.66	236.21	NA	238.00
GISS-E2-R	240.26	239.02	NA	NA	NA	240.75
HadGEM2	242.92	241.58	241.8	241.02	242.76	NA
HadGEM3	238.54	237.58	NA	NA	NA	NA
MIROC-SPRINTARS	235.62	234.72	234.81	234.45	235.69	NA

NCAR-CESM1-CAM4	233.15	232.05	232.24	232.14	NA	233.76
NCAR-CESM1-CAM5	233.65	232.74	NA	NA	NA	NA
NorESM1	234.13	233.04	NA	233.21	234.43	234.61
			**rlds**			
ECHAM-HAM	348.57	349.76	349.55	349.64	NA	349.13
GISS-E2-R	348.93	349.86	NA	NA	NA	349.44
HadGEM2	333.28	334.01	333.81	334.04	332.69	NA
HadGEM3	337.56	338.75	NA	NA	NA	NA
MIROC-SPRINTARS	345.46	346.39	346.29	346.23	345.54	NA
NCAR-CESM1-CAM4	338.99	340.12	339.82	339.79	NA	339.85
NCAR-CESM1-CAM5	343.66	344.60	NA	NA	NA	NA
NorESM1	342.27	343.66	NA	343.28	342.51	343.10
			**rlus**			

ECHAM-HAM	400.18	400.56	400.58	400.54	NA	400.01
GISS-E2-R	405.45	405.64	NA	NA	NA	405.52
HadGEM2	395.68	395.03	394.98	395.18	394.67	NA
HadGEM3	397.22	397.68	NA	NA	NA	NA
MIROC-SPRINTARS	398.47	398.75	398.71	398.90	398.53	NA
NCAR-CESM1-CAM4	397.49	397.78	397.66	397.74	NA	397.57
NCAR-CESM1-CAM5	396.03	396.45	NA	NA	NA	NA
NorESM1	400.61	401.08	NA	401.02	401.14	400.41
**Coupled:**			**rlut**			
ECHAM-HAM	238.49	239.05	238.93	238.88	NA	238.33
GISS-E2-R	240.37	239.93	NA	NA	NA	241.63
HadGEM2	243.09	243.12	243.46	243.53	243.78	NA
HadGEM3	243.33	245.03	NA	NA	NA	NA
MIROC-SPRINTARS	233.32	233.47	233.51	233.3	233.37	NA

NCAR-CESM1-CAM4	238.00	239.23	NA	238.85	NA	239.09
NCAR-CESM1-CAM5	237.00	237.78	NA	NA	NA	NA
NorESM1	232.08	232.50	NA	232.35	232.35	233.08
			**rlds**			
ECHAM-HAM	349.54	356.17	355.01	356.08	NA	349.63
GISS-E2-R	350.24	353.61	NA	NA	NA	352.99
HadGEM2	333.15	339.14	339.65	342.54	335.02	NA
HadGEM3	349.98	358.71	NA	NA	NA	NA
MIROC-SPRINTARS	337.31	341.38	340.98	341.3	337.49	NA
NCAR-CESM1-CAM4	352.65	361.93	NA	359.98	NA	355.29
NCAR-CESM1-CAM5	349.81	356.10	NA	NA	NA	NA
NorESM1	334.85	341.08	NA	339.71	335.23	337.87
			**rlus**			

ECHAM-HAM	401.48	406.36	405.57	406.52	NA	400.65
GISS-E2-R	406.4	408.56	NA	NA	NA	408.22
HadGEM2	395.66	399.91	400.51	403.04	397.23	NA
HadGEM3	408.59	415.49	NA	NA	NA	NA
MIROC-SPRINTARS	393.96	396.63	396.43	397.00	394.06	NA
NCAR-CESM1-CAM4	409.53	416.68	NA	415.27	NA	410.89
NCAR-CESM1-CAM5	402.64	407.37	NA	NA	NA	NA
NorESM1	394.92	399.47	NA	398.59	395.76	397.24

Table S16 phase 2: Global and annual multi-model mean radiation fluxes *(no data for CanESM2, IPSL-CM5A or MPI-SM available).

Model	Base	cfc12	cfc11	n2o1p	Indus	bcslt
**FSST:**			**hfss**			
ECHAM-HAM	23.394	23.302	23.4	23.213	NA	22.155
GISS-E2-R	19.293	19.1	NA	NA	NA	18.136

HadGEM2	18.372	17.657	17.694	17.528	17.954	NA
HadGEM3	18.649	18.643	NA	NA	NA	NA
MIROC-SPRINTARS	14.766	14.828	14.771	14.629	14.744	NA
NCAR-CESM1-CAM4	17.553	17.608	17.533	17.477	NA	16.434
NCAR-CESM1-CAM5	17.259	17.328	NA	NA	NA	NA
NorESM1	18.459	18.538	NA	18.393	18.692	17.265
			**hfls**			
ECHAM-HAM	86.320	85.656	85.517	85.265	NA	84.837
GISS-E2-R	93.687	93.369	NA	NA	NA	91.928
HadGEM2	89.372	90.297	90.338	89.764	90.348	NA
HadGEM3	88.713	88.394	NA	NA	NA	NA
MIROC-SPRINTARS	94.490	94.152	94.209	93.761	94.610	NA
NCAR-CESM1-CAM4	81.687	81.406	81.457	81.300	NA	80.109

NCAR-CESM1-CAM5	87.405	86.910	NA	NA	NA	NA
NorESM1	83.120	82.853	NA	82.854	82.931	81.600
**Coupled:**			**hfss**			
ECHAM-HAM	23.467	23.140	23.167	23.002	NA	22.256
GISS-E2-R	18.878	18.669	NA	NA	NA	17.779
HadGEM2	18.523	18.222	18.190	17.919	18.736	NA
HadGEM3	18.157	17.925	NA	NA	NA	NA
MIROC-SPRINTARS	14.358	14.295	14.297	14.063	14.296	NA
NCAR-CESM1-CAM4	17.457	17.162	NA	17.152	NA	16.300
NCAR-CESM1-CAM5	17.085	16.753	NA	NA	NA	NA
NorESM1	18.692	18.592	NA	18.533	19.112	17.771
			**hfls**			
ECHAM-HAM	86.475	88.141	87.767	87.808	NA	84.744

GISS-E2-R	94.444	95.198	NA	NA	NA	93.728
HadGEM2	89.316	90.606	90.849	91.213	90.109	NA
HadGEM3	93.450	96.087	NA	NA	NA	NA
MIROC-SPRINTARS	91.379	92.421	92.336	92.056	91.550	NA
NCAR-CESM1-CAM4	86.850	89.715	NA	88.948	NA	86.054
NCAR-CESM1-CAM5	89.984	92.009	NA	NA	NA	NA
NorESM1	80.075	81.739	NA	81.249	79.512	79.839

Table S17 phase 2: Global and annual multi-model mean temperature, precipitation and evaporation (no data for CanESM2, IPSL-CM5A or MPI-ESM available and no evspsbl data for HadGEM3).

Model	Base	cfc12	cfc11	n2o1p	Indus	bcslt
**FSST:**			**tas**			
ECHAM-HAM	287.87	287.96	287.95	287.95	NA	288.19
GISS-E2-R	289.05	289.10	NA	NA	NA	289.08
HadGEM2	286.79	286.73	286.72	286.78	286.65	NA

HadGEM3	287.30	287.39	NA	NA	NA	NA
MIROC-SPRINTARS	288.09	288.14	288.13	288.19	288.11	NA
NCAR-CESM1-CAM4	287.29	287.35	287.33	287.34	NA	287.35
NCAR-CESM1-CAM5	286.89	286.97	NA	NA	NA	NA
NorESM1	287.75	287.85	NA	287.85	287.84	287.77
			**pr**			
ECHAM-HAM	3.4586E-05	3.4310E-05	3.4261E-05	3.4164E-05	NA	3.3990E-05
GISS-E2-R	3.7473E-05	3.7346E-05	NA	NA	NA	3.6770E-05
HadGEM2	3.5725E-05	3.6095E-05	3.6112E-05	3.5881E-05	3.6115E-05	NA
HadGEM3	3.5503E-05	3.5377E-05	NA	NA	NA	NA
MIROC-SPRINTARS	3.7797E-05	3.7660E-05	3.7682E-05	3.7503E-05	3.7842E-05	NA
NCAR-CESM1-CAM4	3.2654E-05	3.2541E-05	3.2562E-05	3.2500E-05	NA	3.2024E-05
NCAR-CESM1-CAM5	3.4961E-05	3.4762E-05	NA	NA	NA	NA

NorESM1	3.3229E-05	3.3124E-05	NA	3.3119E-05	3.3152E-05	3.2618E-05
			**evspsbl**			
ECHAM-HAM	-3.4500E-05	-3.4234E-05	-3.4178E-05	-3.4077E-05	NA	-3.3906E-05
GISS-E2-R	3.7475E-05	3.7347E-05	NA	NA	NA	3.6771E-05
HadGEM2	3.5700E-05	3.6071E-05	3.6088E-05	3.5858E-05	3.6091E-05	NA
MIROC-SPRINTARS	3.7796E-05	3.7661E-05	3.7683E-05	3.7504E-05	3.7844E-05	NA
NCAR-CESM1-CAM4	3.2655E-05	3.2542E-05	3.2563E-05	3.2500E-05	NA	3.2024E-05
NCAR-CESM1-CAM5	3.4962E-05	3.4764E-05	NA	NA	NA	NA
NorESM1	3.3227E-05	3.3121E-05	NA	3.3122E-05	3.3153E-05	3.2619E-05
**Coupled:**			**tas**			
ECHAM-HAM	288.15	289.10	288.95	289.14	NA	288.01
GISS-E2-R	289.23	289.63	NA	NA	NA	289.57
HadGEM2	286.75	287.56	287.69	288.18	287.08	NA
HadGEM3	289.41	290.68	NA	NA	NA	NA

MIROC-SPRINTARS	287.40	287.89	287.85	287.99	287.43	NA
NCAR-CESM1-CAM4	289.52	290.88	NA	290.62	NA	289.81
NCAR-CESM1-CAM5	288.18	289.09	NA	NA	NA	NA
NorESM1	286.69	287.58	NA	287.41	286.82	287.19
			**pr**			
ECHAM-HAM	3.4634E-05	3.5309E-05	3.5159E-05	3.5177E-05	NA	3.394E-05
GISS-E2-R	3.7776E-05	3.8078E-05	NA	NA	NA	3.7490E-05
HadGEM2	3.5704E-05	3.6220E-05	3.6318E-05	3.6462E-05	3.6023E-05	NA
HadGEM3	3.7415E-05	3.8478E-05	NA	NA	NA	NA
MIROC-SPRINTARS	3.6551E-05	3.6968E-05	3.6934E-05	3.6822E-05	3.6620E-05	NA
NCAR-CESM1-CAM4	3.4718E-05	3.5864E-05	NA	3.5558E-05	NA	3.4400E-05
NCAR-CESM1-CAM5	3.5993E-05	3.6802E-05	NA	NA	NA	NA
NorESM1	3.2005E-05	3.2672E-05	NA	3.2476E-05	3.1782E-05	3.1912E-05

			evspsbl			
ECHAM-HAM	-3.4557E-05	-3.5225E-05	-3.5076E-05	-3.5092E-05	NA	-3.3864E-05
GISS-E2-R	3.7778E-05	3.8079E-05	NA	NA	NA	3.7491E-05
HadGEM2	3.5679E-05	3.6196E-05	3.6294E-05	3.6440E-05	3.6000E-05	NA
MIROC-SPRINTARS	3.6552E-05	3.6969E-05	3.6934E-05	3.6822E-05	3.6620E-05	NA
NCAR-CESM1-CAM4	3.4718E-05	3.5865E-05	NA	3.5558E-05	NA	3.4400E-05
NCAR-CESM1-CAM5	3.5994E-05	3.6804E-05	NA	NA	NA	NA
NorESM1	3.2006E-05	3.2673E-05	NA	3.2477E-05	3.1782E-05	3.1913E-05

Table S18 phase 2: Global and annual multi-model mean humidity and pressure *(no data for CanESM2, IPSL-CM5A or MPI-ESM available. Data also missing for HadGEM3 for fsst huss and hurs).

Model	Base	cfc12	cfc11	n2o1p	Indus	bcslt
FSST:			huss			
ECHAM-HAM	0.0097324	0.00047691	0.00047604	0.00047781	NA	0.0097894
GISS-E2-R	0.010336	0.010378	NA	NA	NA	0.010444

HadGEM2	0.0094261	0.0095636	0.0095646	0.0095842	0.0095225	NA
HadGEM3	0.0096841	0.009722	NA	NA	NA	NA
MIROC-SPRINTARS	0.0095515	0.0095723	0.0095732	0.0095973	0.0095526	NA
NCAR-CESM1-CAM4	0.010341	0.010358	0.010361	0.010371	NA	0.010454
NCAR-CESM1-CAM5	0.010131	0.010152	NA	NA	NA	NA
NorESM1	0.010468	0.010495	NA	0.01051	0.010466	0.010600
			**hurs**			
ECHAM-HAM	75.151	53.033	53.052	52.887	NA	74.870
GISS-E2-R	NA	73.904	NA	NA	NA	74.211
HadGEM2	76.055	76.967	76.955	76.959	76.925	NA
MIROC-SPRINTARS	77.667	77.642	77.681	77.706	77.678	NA
NCAR-CESM1-CAM4	79.324	79.243	79.307	79.323	NA	79.708
NCAR-CESM1-CAM5	78.803	78.684	NA	NA	NA	NA

NorESM1	78.252	78.169	NA	78.252	78.038	78.798
			**ps**			
ECHAM-HAM	98550	98550	98550	98550	NA	98550
GISS-E2-R	98400	98400	NA	NA	NA	98400
HadGEM2	98572	98574	98574	98573	98572	NA
HadGEM3	98522	98523	NA	NA	NA	NA
MIROC-SPRINTARS	98681	98682	98682	98682	98681	NA
NCAR-CESM1-CAM4	98541	98541	98541	98541	NA	98544
NCAR-CESM1-CAM5	98541	98542	NA	NA	NA	NA
NorESM1	98546	98547	NA	98547	98546	98549
**Coupled:**			**huss**			
ECHAM-HAM	0.0097884	0.00053711	0.00052578	0.00053786	NA	0.0098089
GISS-E2-R	0.010476	0.010764	NA	NA	NA	0.010814
HadGEM2	0.0094013	0.0098433	0.0098935	0.01015	0.0096048	NA

HadGEM3	NA	0.011642	NA	NA	NA	NA
MIROC-SPRINTARS	0.0088505	0.0091385	0.0091122	0.0091746	0.0088678	NA
NCAR-CESM1-CAM4	0.011606	0.012411	NA	0.012256	NA	0.01191
NCAR-CESM1-CAM5	0.010653	0.0112	NA	NA	NA	NA
NorESM1	0.0098209	0.010262	NA	0.010184	0.0098127	0.010097
			**hurs**			
ECHAM-HAM	74.789	52.25	52.283	52.038	NA	75.239
GISS-E2-R	74.017	74.09	NA	NA	NA	74.456
HadGEM2	75.987	75.976	75.953	75.922	76.098	NA
HadGEM3	75.899	75.799	NA	NA	NA	NA
MIROC-SPRINTARS	77.490	77.536	77.563	77.627	77.708	77.605
NCAR-CESM1-CAM4	78.692	78.577	NA	78.651	NA	79.095
NCAR-CESM1-CAM5	78.150	78.171	NA	NA	NA	NA

NorESM1	78.304	78.142	NA	78.212	77.898	78.394
			ps			
ECHAM-HAM	98550	98550	98550	98550	NA	98550
GISS-E2-R	98400	98400	NA	NA	NA	98400
HadGEM2	98571	98587	98588	98596	98578	NA
HadGEM3	98553	98575	NA	NA	NA	NA
MIROC-SPRINTARS	98655	98664	98663	98664	98655	NA
NCAR-CESM1-CAM4	98579	98603	NA	98598	NA	98588
NCAR-CESM1-CAM5	98554	98570	NA	NA	NA	NA
NorESM1	98525	98538	NA	98535	98525	98532

Table S19: Global and annual multi-model mean precipitation and cloud cover *(no data for CanESM2, IPSL-CM5A or MPI-ESM and no data available for clt for NCAR-CESM1-CAM5 or for prw for HadGEM3).

**Model**	**Base**	**cfc12**	**cfc11**	**n2o1p**	**Indus**	**bcslt**
**FSST:**			**prc**			

ECHAM-HAM	2.3107E-05	2.3016E-05	2.2944E-05	2.2845E-05	NA	2.2641E-05
GISS-E2-R	1.5405E-05	1.5349E-05	NA	NA	NA	1.5065E-05
HadGEM2	2.6590E-05	2.6861E-05	2.6877E-05	2.6701E-05	2.6926E-05	NA
HadGEM3	2.5862E-05	2.5738E-05	NA	NA	NA	NA
MIROC-SPRINTARS	1.9175E-05	1.9151E-05	1.9136E-05	1.9034E-05	1.9214E-05	NA
NCAR-CESM1-CAM4	2.0003E-05	1.9919E-05	1.9952E-05	1.9947E-05	NA	1.9509E-05
NCAR-CESM1-CAM5	2.4777E-05	2.4655E-05	NA	NA	NA	NA
NorESM1	2.0252E-05	2.0152E-05	NA	2.0162E-05	2.0158E-05	1.9772E-05
			**prw**			
ECHAM-HAM	26.482	26.568	26.510	26.577	NA	26.608
GISS-E2-R	24.882	24.992	NA	NA	NA	25.066
HadGEM2	22.174	22.434	22.434	22.438	22.304	NA
MIROC-SPRINTARS	25.118	25.185	25.183	25.192	25.123	NA

NCAR-CESM1-CAM4	25.519	25.572	25.560	25.579	NA	25.812
NCAR-CESM1-CAM5	25.561	25.614	NA	NA	NA	NA
NorESM1	26.032	26.115	NA	26.140	26.021	26.347
			**clt**			
ECHAM-HAM	68.114	67.939	68.115	68.221	NA	68.261
GISS-E2-R	61.180	60.983	NA	NA	NA	60.922
HadGEM2	53.413	53.414	53.312	53.249	53.561	NA
HadGEM3	64.458	64.141	NA	NA	NA	NA
MIROC-SPRINTARS	67.906	67.443	67.523	67.792	67.914	NA
NCAR-CESM1-CAM4	53.874	53.431	53.551	53.659	NA	54.209
NorESM1	54.383	54.146	NA	54.311	54.365	54.786
**Coupled:**			**prc**			
ECHAM-HAM	2.3274E-05	2.3895E-05	2.3768E-05	2.3776E-05	NA	2.2580E-05

GISS-E2-R	1.5649E-05	1.6001E-05	NA	NA	NA	1.5694E-05
HadGEM2	2.6575E-05	2.6955E-05	2.7064E-05	2.7197E-05	2.6851E-05	NA
HadGEM3	2.7617E-05	2.8543E-05	NA	NA	NA	NA
MIROC-SPRINTARS	1.8624E-05	1.8855E-05	1.8844E-05	1.8825E-05	1.8666E-05	NA
NCAR-CESM1-CAM4	2.1509E-05	2.2575E-05	NA	2.2319E-05	NA	2.1281E-05
NCAR-CESM1-CAM5	2.5977E-05	2.6545E-05	NA	NA	NA	NA
NorESM1	1.9619E-05	2.0043E-05	NA	1.9932E-05	1.9474E-05	1.9451E-05
			**prw**			
ECHAM-HAM	26.623	28.344	28.029	28.393	NA	26.652
GISS-E2-R	25.286	26.204	NA	NA	NA	26.233
HadGEM2	22.089	23.427	23.562	24.249	22.702	NA
MIROC-SPRINTARS	22.442	23.344	23.252	23.361	22.487	NA
NCAR-CESM1-CAM4	29.383	31.844	NA	31.331	NA	30.320

NCAR-CESM1-CAM5	26.875	28.541	NA	NA	NA	NA
NorESM1	23.910	25.244	NA	24.981	23.901	24.643
			**clt**			
ECHAM-HAM	67.708	67.112	67.205	67.17	NA	68.26
GISS-E2-R	61.223	60.957	NA	NA	NA	60.880
HadGEM2	53.476	53.292	53.209	53.046	53.635	NA
HadGEM3	62.856	61.805	NA	NA	NA	NA
MIROC-SPRINTARS	66.947	66.586	66.619	66.837	66.976	NA
NCAR-CESM1-CAM4	53.578	53.405	NA	53.627	NA	54.261
NorESM1	53.461	53.363	NA	53.535	53.451	53.786

Table S20: Global and annual multi-model mean wind *(no data for CanESM2, IPSL-CM5A or MPI-ESM, HadGEM3 data only for coupled experiments).

Model	Base	cfc12	cfc11	n2o1p	Indus	bcslt
**FSST:**			**uas**			

ECHAM-HAM	-0.41503	-0.42614	-0.39907	-0.37536	NA	-0.42886
GISS-E2-R	-0.11532	-0.12117	NA	NA	NA	-0.13115
HadGEM2	-0.42558	-0.41170	-0.42870	-0.39732	-0.38772	NA
HadGEM3	-0.50725	-0.5197	NA	NA	NA	NA
MIROC-SPRINTARS	-0.42289	-0.44763	-0.42295	-0.43185	-0.43115	NA
NCAR-CESM1-CAM4	-0.7745	-0.77007	-0.77927	-0.7744	NA	-0.77336
NCAR-CESM1-CAM5	-0.71676	-0.73421	NA	NA	NA	NA
NorESM1	-0.79522	-0.79502	NA	-0.77465	-0.76785	-0.79769
			**vas**			
ECHAM-HAM	0.25979	0.25394	0.26370	0.23723	NA	0.25659
GISS-E2-R	0.10155	0.10164	NA	NA	NA	0.11095
HadGEM2	0.15878	0.15990	0.16025	0.15146	0.15867	NA
HadGEM3	0.13992	0.13875	NA	NA	NA	NA
MIROC-SPRINTARS	0.16342	0.17083	0.15887	0.16131	0.17360	NA

NCAR-CESM1-CAM4	0.15064	0.14447	0.13923	0.14024	NA	0.17368
NCAR-CESM1-CAM5	0.17138	0.18079	NA	NA	NA	NA
NorESM1	0.083783	0.076473	NA	0.087951	0.085757	0.096464
**Coupled:**			**uas**			
ECHAM-HAM	-0.38785	-0.35994	-0.35776	-0.35961	NA	-0.39413
GISS-E2-R	-0.12145	-0.12493	NA	NA	NA	-0.14329
HadGEM2	-0.40216	-0.39952	-0.38174	-0.36890	-0.35231	NA
HadGEM3	-0.43758	-0.43044	NA	NA	NA	NA
MIROC-SPRINTARS	-0.44940	-0.43917	-0.44147	-0.42975	-0.44765	NA
NCAR-CESM1-CAM4	-0.62346	-0.62313	NA	-0.62279	NA	-0.67841
NCAR-CESM1-CAM5	-0.50129	-0.46520	NA	NA	NA	NA
NorESM1	-0.73082	-0.71637	NA	-0.71739	-0.72721	-0.70018
			**vas**			

ECHAM-HAM	0.25157	0.26221	0.25538	0.26624	NA	0.24636
GISS-E2-R	0.099954	0.10111	NA	NA	NA	0.12079
HadGEM2	0.13763	0.14569	0.14257	0.13592	0.14656	NA
HadGEM3	0.02419	0.024334	NA	NA	NA	NA
MIROC-SPRINTARS	0.13726	0.13705	0.1378	0.13292	NA	NA
NCAR-CESM1-CAM4	0.005655	-0.017541	NA	-0.0062849	NA	0.034311
NCAR-CESM1-CAM5	0.046967	0.024539	NA	NA	NA	NA
NorESM1	0.046196	0.05255	NA	0.056392	0.053092	0.048323

[i] HadGEM3 omitted from cfc12 means because it lacks data for the last month
